# AHCYL1 mediates the tumor-promoting effect of PREX2 in non-small cell lung carcinoma

**DOI:** 10.7150/thno.108654

**Published:** 2025-04-21

**Authors:** Mingjuan Lei, Yiu To Yeung, Wenna Nie, Ran Yang, Jian Li, Hanyong Chen, Ran Zhao, Kangdong Liu, Zigang Dong

**Affiliations:** 1 Department of Pathophysiology, School of Basic Medical Sciences, Zhengzhou University, Zhengzhou, Henan, China.; 2 China-US (Henan) Hormel Cancer Institute, Zhengzhou, Henan, China.; 3 The Hormel Institute, University of Minnesota, MN, USA.

**Keywords:** Phosphatidylinositol-3,4,5-trisphosphate-dependent Rac exchanger factor 2 (PREX2), Adenosylhomocysteinase Like 1 (AHCYL1), GEF activity, Cell growth, NSCLC

## Abstract

**Rationale:** As the most common form of lung cancer, non-small cell lung cancer (NSCLC) is still a challenging disease. Even though molecular-targeted drugs have greatly benefited NSCLC patients, the limited number of effective targets and the emergence of drug resistance necessitate further research to identify new candidates and improve clinical outcomes. Phosphatidylinositol-3,4,5-triphosphate-dependent RAC exchange factor-2 (PREX2) is highly expressed in multiple cancer types and poses high mutation frequency in lung cancer. However, the study of PREX2 in lung cancer, especially NSCLC, is few and unclear, thus, the role of PREX2 and the regulatory mechanism of PREX2 in NSCLC is worthy of further investigation.

**Methods:** To determine the tumor-promoting effects of PREX2 in NSCLC, we established PREX2 knockdown NSCLC cells, then assessed cell growth in *vitro* and in cell-derived xenograft (CDX) mouse model. Furtherly, we used the urethane-induced lung carcinogenesis mouse model to confirm the significance of PREX2 *in vivo*. Additionally, we identified AHCYL1 as a novel PREX2-interacting protein through pull-down assay and liquid chromatography with tandem mass spectrometry (LC-MS/MS) and investigated the mechanisms of PREX2 GEF activity regulated by AHCYL1 using various molecular biology assays, including western blotting, *in vitro* GEF assay and active RAC1 pull-down assay.

**Results:** Our study suggests that PREX2 and AHCYL1 both promote NSCLC cell growth and proves that AHCYL1 enhances the GEF activity of PREX2 by alleviating the mutual inhibition between PREX2 and PTEN. Consequently, AHCYL1 intensifies the tumor-promoting effects of PREX2 in NSCLC.

**Conclusion:** Overall, our results indicate that PREX2 and AHCYL1 promote lung cancer development and reveal a novel regulatory mechanism of PREX2 GEF activity by AHCYL1, which will contribute to the understanding of NSCLC pathogenesis and offer new targets and strategies for the diagnosis and treatment of NSCLC.

## Introduction

Lung cancer is the most common malignancy and the leading cause of cancer mortality 1, 2. As the most common form of lung cancer, non-small cell lung cancer (NSCLC) is usually diagnosed at an advanced stage. The poor prognosis and resistance to both radiation and chemotherapy of NSCLC lead to its 5-year overall survival rate as low as approximately 20% 3. Over the last two decades, many oncogenic drivers, such as epidermal growth factor receptor (EGFR), anaplastic lymphoma kinase (ALK), v-raf murine sarcoma viral oncogene homolog B (BRAF), ROS proto-oncogene 1 (ROS1), Kirstin rat sarcoma virus (KRAS), human epidermal receptor 2 (HER2), c-MET proto-oncogene (MET), have been identified in approximately 60% of lung adenocarcinoma patients in Western populations and 80% among Asian population 4. Precision therapies targeting these driver genes, such as Epidermal growth factor receptor tyrosine kinase inhibitors (EGFR-TKIs), have shown considerable benefits in NSCLC patients 5. Yet, major challenges remain, including identifying additional actionable targets to expand the population that benefits from targeted therapies, and a better understanding of NSCLC development and progression mechanisms to overcome the resistance to common therapies 6, 7. Therefore, elucidating molecular mechanisms underlying the initiation and progression of lung cancer and identifying novel therapeutic targets are still of great clinical importance.

Phosphatidylinositol-3,4,5-trisphosphate-dependent Rac exchanger factor 2 (PREX2) was originally identified as a guanine nucleotide exchange factor (GEF) for the small GTPase, Rac. Upon the stimulation of phosphatidylinositol 3,4,5-trisphosphate (PIP_3_) and the βγ subunits of heterotrimeric G proteins (G_βγ_), PREX2 exerts its GEF activity to promote the dissociation of GDP from Rac and free GTP binding with Rac 8, 9. The active Rac GTPase can regulate a wide range of cell functions, ranging from cytoskeleton-linked aspects such as cell growth and cell movement to others such as transcription or the production of reactive oxygen species 10, 11. The PREX2 locus is in a genomic region, chromosome 8q13, which is usually linked to gene amplification and aggressive cancer phenotypes 12, 13. Manipulation of PREX2 expression could alter several pivotal cellular activities of tumor cells, such as apoptosis, proliferation, and migration in various cancers 14-19. In these previous studies, PREX2 has been considered as a tumor-promoting gene because of its GEF activity toward Rac GTPase and its inhibition of the tumor suppressor PTEN, a lipid phosphatase that antagonizes PI3K by dephosphorylating PIP3, therefore reducing AKT activation 20-23. Furthermore, stringent analysis of combinations of databases revealed somatic mutations of PREX2 in an average of 3% of samples from different types of human cancers 24. Skin and lung cancers represent the tumors that most frequently bear PREX2 mutations. PREX2 is the third most mutated protein in melanoma after B-Raf and N-Ras and among the 10 most frequently mutated genes in lung cancer 25, 26. The mutations are distributed throughout the protein and appear to play a more consistent role in promoting cancer cell proliferation, survival, anchorage-independent cell growth, and xenograft tumor growth in different cancer types 17, 21, 27. The tumor-promoting effect and high mutation frequency of PREX2 make it an attractive target for cancer therapy. However, the study of PREX2 in lung cancer, especially NSCLC is few and unclear, thus, the role of PREX2 and the regulatory mechanism of PREX2 in NSCLC is worthy of further investigation.

In this work, we studied the functional and cellular regulatory characteristics of PREX2 in NSCLC. We found that PREX2 was frequently upregulated in NSCLC cell lines and patient tissues. Silencing of PREX2 inhibited cell growth of NSCLC cells and significantly suppressed tumor growth of NSCLC cell-derived xenografts (CDX). And *Prex2* knockout mice showed less susceptibility to urethane induction of lung carcinogenesis compared with wild-type mice. Furthermore, we identified Adenosylhomocysteinase Like 1 (AHCYL1, also called IRBIT) as a novel PREX2-interacting protein, which could also regulate cell growth in NSCLC cells and CDX model. Mechanistically, we demonstrated that AHCYL1 could release PTEN inhibition by competitively binding the PDZ2 domain of PREX2 and enhance the GEF activity of PREX2. Therefore, AHCYL1 mediated the tumor-promoting effect of PREX2 in NSCLC cells. Overall, our findings suggested that PREX2 and AHCYL1 played important roles in lung cancer and illustrated a novel regulatory mechanism of PREX2 function by AHCYL1.

## Results

### PREX2 is frequently upregulated in human NSCLC and promotes NSCLC cell growth *in vitro* and* in vivo*

**Figure 1:**
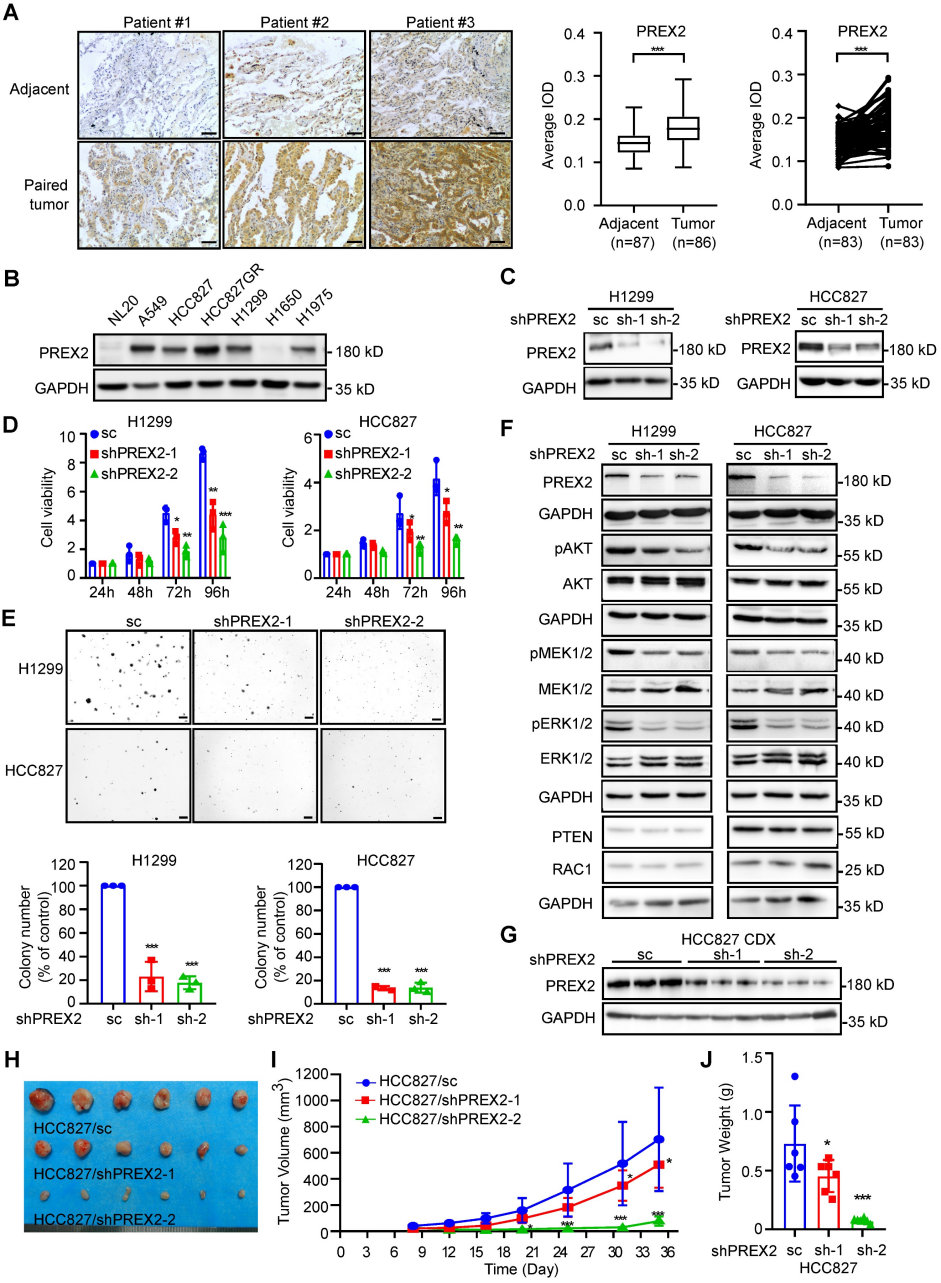
** PREX2 was upregulated in human NSCLC and promoted cell growth *in vitro* and* in vivo*. A**. The expression of PREX2 was examined by immunohistochemistry (IHC) staining using a lung cancer tissue array. The left panels show representative images of paired lung cancer tissues and adjacent tissues. Scale bar:100 μm. The right panels show quantification of all samples (adjacent tissues: n=87; tumor tissues: n=86) and paired samples (n=83). The statistical significance was determined by a two-tailed paired t test. ***, p < 0.001. **B.** The expression of PREX2 in human normal lung epithelial cell NL20 and NSCLC cell lines was measured by western blotting. **C.** The level of PREX2 was measured in H1299 and HCC827 cells after knocking down by lentiviral transduction (sc: scrambled negative control of shRNA; shPREX2-1 and shPREX2-2). **D.** Cell viability of PREX2 knockdown NSCLC cells after cell seeding for 24, 48, 72 and 96 h was determined by MTT assay. Data are presented as means ± SD from 3 independent experiments (n = 3). Statistical significance was determined using multiple t-test. *, p < 0.05. **, p < 0.01. ***, p < 0.001. **E.** Anchorage-independent growth of NSCLC cells with PREX2 knockdown was determined by soft agar assay. Representative images are shown in the upper panels. Colonies with a diameter more than 50 μm were counted using Image-Pro Plus (v.6) computer software and normalized with control group. Data are presented as means ± SD from 3 independent experiments (n=3). The statistical analysis was determined using one-way ANOVA. Scale bar: 200 μm. ***, p < 0.001. **F.** The MEK/ERK and PI3K/AKT signaling pathways were detected by western blotting after PREX2 knockdown in H1299 and HCC827 cells. pAKT (Ser473), pMEK1/2 (Ser217/221), and pERK1/2 (Thr202/Tyr204) were measured. **G.** The level of PREX2 in the tumor tissues collected from control and PREX2 knockdown cells injected mice was detected by western blotting. **H.** The tumors collected from control and PREX2 knockdown cells injected mice are shown (n=6). **I.** The longitudinal tumor volume in the control and PREX2 knockdown mice groups were measured and calculated every 3-4 days. Data are presented as means ± SD (n=6). Statistical significance was determined using one-way ANOVA. *, p < 0.05. ***, p < 0.001. **J.** The final tumor weights in the control and PREX2 knockdown mice groups were measured at the endpoints. Data are presented as means ± SD (n=6). Statistical significance was determined using one-way ANOVA. *, p < 0.05. ***, p < 0.001.

To elucidate the expression level of PREX2 in NSCLC, we performed immunohistochemistry (IHC) analysis with 90 paired human lung cancer and matched adjacent tissues. PREX2 level was significantly higher in tumor tissues compared with adjacent tissues (Figure 1A). The representative images of paired tissues are shown. While the expression level of PREX2 of patients in clinical stage 3 and Grade III was higher than that of patients in other stages or grades, there was no significant difference between tumors across ages (Figure S1A). Kaplan-Meier analysis showed no difference in survival probability between patients with high and low levels of PREX2 (Figure S1B). The PREX2 locus is in a genomic region, chromosome 8q13, which is usually linked to gene amplification. By analyzing the TCGA cohort, we found that PREX2 RNA abundance is correlated with PREX2 copy numbers except in those lung adenocarcinoma (LUAD) patients with high expression of PREX2 (Figure S1C). And differential prognostic implications of PREX2 are observed in distinct NSCLC patient subgroups. The Kaplan-Meier analysis showed that high copy number and high expression of PREX2 in lung squamous cell carcinoma (LUSC) patients had a significantly shorter survival time but not in LUAD patients (Figure S1D-E). We expanded the bioinformatic analysis with the Kaplan-Meier Plotter, which integrates multiple datasets (including the TCGA cohort) to assess overall trends 28. The analysis revealed that high PREX2 expression is strongly correlated with poor prognosis in LUAD patients (Figure S1F). The seemingly differential results may reflect the complexity of cancer biology and clinical research, requiring deeper investigation and further experimental validation. We also determined the protein level of PREX2 in NSCLC cell lines. Results indicated that increased expression of PREX2 occurs in 5 out of 6 human NSCLC cell lines compared with normal lung epithelial cell line NL20 (Figure 1B). The relatively higher expression of PREX2 in lung cancer tissues and NSCLC cell lines suggests that dysregulation of PREX2 may play a role in NSCLC.

To determine the biological significance of PREX2 in NSCLC, we knocked down PREX2 expression by two different shPREX2 sequences in H1299 and HCC827 (Figure 1C). Silencing of PREX2 in NSCLC cells significantly inhibited cell proliferation and anchorage-independent cell growth (Figure 1D-E). Due to its Rac regulatory properties, PREX2 knockdown cells appeared to be larger (Figure S2A). The cell migration and invasive capacity were attenuated in PREX2 knockdown cells (Figure S2B-C). Because of the low transfection efficiency of the full-length PREX2 (183 kD), we overexpressed the PREX2 catalytic domains (DH-PH) instead and observed the promotion of the anchorage-independent cell growth by DH-PH overexpression in NSCLC cells (Figure S3A-C). Furtherly, we checked the phosphorylation of ERK1/2, an effector of PREX2 homology PREX1 downstream from RAC1 29, 30, and AKT, downstream effectors of PTEN 23. The results showed that PREX2 knockdown decreased the phosphorylation levels of ERK1/2, MEK1/2 and AKT (Figure 1F), while overexpression of PREX2 catalytic DH-PH domain resulted in elevated phosphorylation of ERK1/2, MEK1/2 and AKT in NSCLC cells (Figure S3D). These results indicate that PREX2 promotes NSCLC cell growth *in vitro* as the GEF factor of RAC1 to mediate the ERK/MEK axis and the suppressor of PTEN to regulate the PI3K/AKT signaling pathway.

To further investigate whether PREX2 regulates NSCLC cell growth *in vivo*, we used cell-derived xenograft (CDX) mouse model and monitored the growth of PREX2 knockdown NSCLC cells injected into the nude mice (Figure 1G). Consistent with the *in vitro* findings, PREX2 downregulation largely attenuated the growth of xenograft tumors. The volume and growth rate of tumors in shPREX2-inoculated mice were significantly decreased compared with the control group (Figure 1H-I). The final average tumor weight in shPREX2 groups was reduced dramatically (Figure 1J). The phosphorylation levels of ERK1/2, MEK1/2 and AKT were also decreased in the shPREX2 inoculated mice group (Figure S4A). These findings suggest that PREX2 promotes aberrant cell growth of NSCLC cells *in vivo*.

### *Prex2*-KO mice were less susceptible to urethane treatment

**Figure 2:**
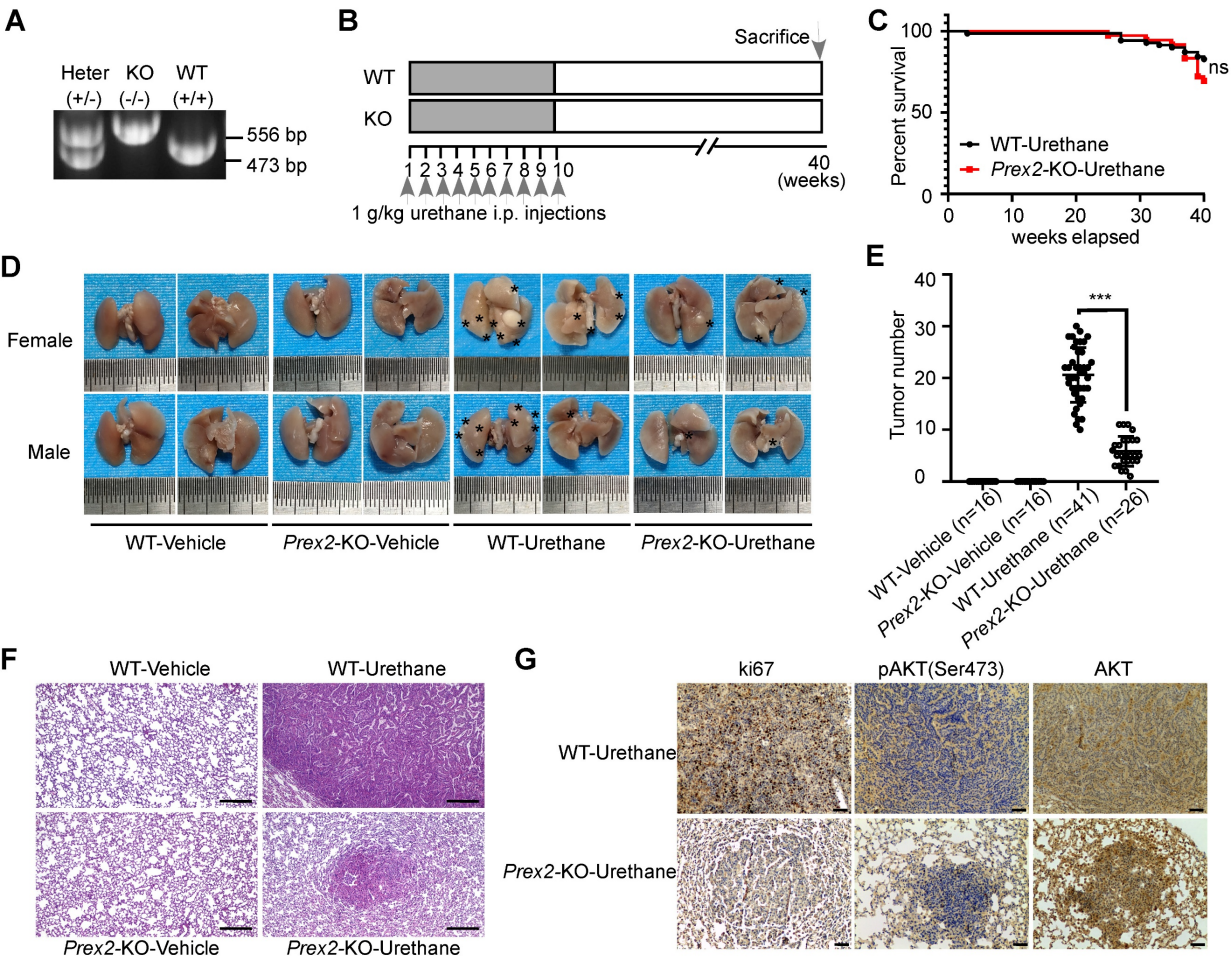
** Prex2-KO mice were less susceptible to urethane treatment. A.** Example of PCR genotyping for wile-type C57BL/6N mice (WT, +/+), *Prex2* knockout mice (KO, -/-) and heterozygous (Heter, +/-) mice are shown. **B.** The schematic diagram for urethane treatment is shown. i.p., intraperitoneally injection. **C.** The effect of *Prex2* knockout on the survival of urethane-treated mice was evaluated by Kaplan-Meier analysis. The statistical analysis was determined using the Mantel-cox test. ns, no significance. **D.** Representative photos of lung samples in different mice groups. The tumor locus was labeled with asterisks. **E.** The tumor numbers of different mice groups were counted at the endpoints. Data are presented as means ± SD (WT-Vehicle: n=16; *Prex2*-KO-Vehicle: n=16; WT-Urethane: n=41; *Prex2*-KO-Urethane: n=26). Statistical significance was determined using one-way ANOVA. ***, p < 0.001. **F.** Lung samples of different mice groups were harvested and stained with H&E. Scale bar: 100 μm. **G.** The expression of ki67, pAKT (Ser473) and total AKT were determined by immunohistochemistry analysis in lungs from urethane-treated WT and *Prex2* knockout mice. Scale bar: 100 μm.

The findings in cell experiments thus far prompted us to investigate the functional significance of PREX2 *in vivo*. Therefore, we used the urethane-induced lung carcinogenesis mouse model, which is well-characterized and accepted as a model for human lung adenocarcinoma, to confirm our hypothesis 31. First, the conventional whole-body *Prex2* knockout mice (*Prex2*-KO) were generated by CRISPR technology at the C57BL/6N genetic background and the *Prex2* knockout homozygotes (*Prex2*-KO) were confirmed by PCR genotyping (Figure 2A). Mice were grouped and treated with urethane according to the schematic diagram shown in Figure 2B. After the urethane treatment, the urethane-treated mice showed signs of illness as evidenced by overall behavior and slightly decreased body weight compared with the vehicle group (Figure S5)**.** The survival rate of urethane-treated WT mice and *Prex2*-KO mice showed no significant difference (Figure 2C). Moreover, a dramatically decreased number of lung tumors occurred in* Prex2*-KO mice compared with WT mice (Figure 2D-E), which suggested that *Prex2*-KO mice were less susceptible to urethane treatment. In addition, the histologic examination after H&E staining showed that the tumors in WT-urethane-treated mice lost normal alveolar architecture and exhibited an increased nuclear/cytoplasmic ratio and cytologic atypia, which were identified as adenomas. However, tumors from *Prex2*-KO-urethane mice displayed only a few adenomas, and lungs from this group retained a majority of the normal alveolar architecture (Figure 2F). In addition, immunohistochemistry data detected reduced positive staining of ki67 and less expression of pAKT (Ser473) in *Prex2* KO mouse lung tissues (Figure 2G). Overall, our findings indicated that mice loss of *Prex2* decreased the sensitivity to urethane-induced lung carcinogenesis. The oncogenic role of PREX2 in NSCLC is supported by this solid *in vivo* evidence.

### AHCYL1 is a novel PREX2-interacting protein and the phosphorylation of AHCYL1 is critical for the interaction

**Figure 3:**
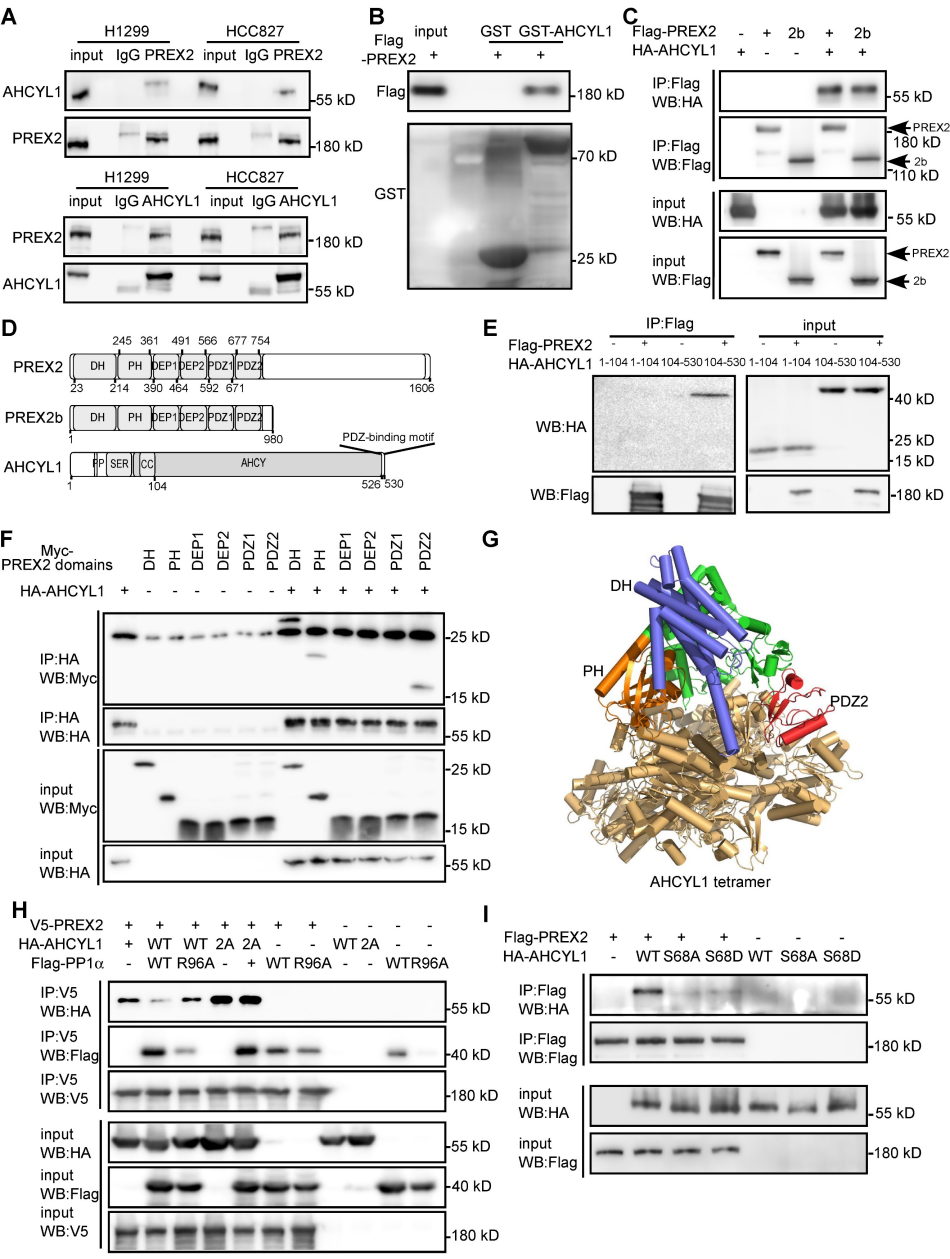
** AHCYL1 is a novel PREX2-interacting protein and the phosphorylation of AHCYL1 is critical for the interaction. A.** Co-immunoprecipitation of endogenous PREX2 and AHCYL1 in H1299 and HCC827 cells was performed with PREX2 antibody (upper panels) or AHCYL1 antibody (lower panels). Immunoprecipitated complexes were detected using AHCYL1 antibody or PREX2 antibody, respectively. Normal IgG is the negative control. **B.** The purified Flag-PREX2 protein was incubated with GST beads or AHCYL1 conjugated-GST beads and detected by Flag antibody. **C.** Co-immunoprecipitation of exogenous HA-AHCYL1 with Flag-PREX2 or Flag-PREX2b in HEK293T cells was performed with Flag antibody and detected by HA antibody. **D.** The domains of PREX2, PREX2b and AHCYL1 are represented in graphical form. For PREX2 and PREX2b, DH, PH, DEP1, DEP2, PDZ1 and PDZ2 domains are indicated. For AHCYL1, the PP1 binding site (PP), the serine-rich region (SER), the coiled-coil domain (CC), and the AHCY domain are indicated. **E.** Co-immunoprecipitation of HA-AHCYL1 truncations (1-104, 104-530) with Flag-PREX2 in HEK293T cells was performed with Flag antibody and detected by HA antibody. **F.** Co-immunoprecipitation of Myc-PREX2 domains (DH, PH, DEP1, DEP2, PDZ1 and PDZ2) with HA-AHCYL1 in HEK293T cells was performed with HA antibody and detected by Myc antibody. **G.** The PREX2 DH-PH-DEP1-DEP2-PDZ1-PDZ2 region and AHCYL1 complex was modeled using AlphaFold3. AHCYL1 was set to 4 copies, based on the reported tetrameric crystal structure (PDB:3MTG) and colored brown yellow. The DH, PH and PDZ2 domain of PREX2 was colored blue, orange and red, respectively. **H.** Co-immunoprecipitation of HA-AHCYL1 wild type (WT) or PP1 non-binding mutant, (I42A/F44A, 2A) with V5-PREX2 in the presence of Flag-PP1α wild type (WT) and phosphatase dead mutant (R96A) in HEK293T cells was performed with V5 antibody and detected by HA antibody. **I.** Co-immunoprecipitation of HA-AHCYL1 wild type (WT), S68A and S68D mutants with Flag-PREX2 in HEK293T cells was performed with Flag antibody and detected by HA antibody.

PREX2 function, especially its GEF activity, is controlled rigidly by its binding partners through conformational or post-translational modifications 17, 20, 32, 33. To investigate the novel regulatory mechanism of PREX2 in NSCLC, we purified PREX2 protein and incubated it with three NSCLC cell lyses. 42 overlapped PREX2-interacting proteins were identified with liquid chromatography with tandem mass spectrometry (LC-MS/MS, Figure S6 and Table S2). AHCYL1 was prioritized based on its high enrichment score in immunoprecipitated PREX2 complexes and its role in mediated Ca^2+^ oscillations, which fine-tune PI3K localization, PI3P-dependent processes (e.g., AKT and mTOR signaling), integrate metabolic and proliferative signals 34. The endogenous interaction between PREX2 and AHCYL1 was validated in NSCLC cell lines, H1299 and HCC827 (Figure 3A). The direct PREX2 and AHCYL1 interaction was confirmed by GST pull-down assay with purified Flag-PREX2 and GST-AHCYL1 recombinant proteins (Figure 3B). Furthermore, the co-immunoprecipitation assay with exogenously expressed proteins in HEK293T cells revealed that both PREX2 and its short isoform PREX2b could interact with AHCYL1 (Figure 3C). Because PREX2 and PREX2b share the same N-terminal, the C-terminal IP4P domain of PREX2 may not be necessary for the interaction. To further identify the key regions responsible for the interaction, we constructed a series of Myc-tagged PREX2 fragments and HA-tagged AHCYL1 fragments for co-IP assays (Figure 3D). The results showed that the C-terminal of AHCYL1 (104-530aa) binds the DH, PH and PDZ2 domain of PREX2 (Figure 3E-F), and the interaction was confirmed with a computational docking model of PREX2 and AHCYL1 (Figure 3G). We isolated several PREX2 mutations that exist in lung cancer through database screening, including P674Q, Q690L, T936A, S926Y, D1256N as well as C243F, which is located in DH-PH domain of PREX2 and exists in NSCLC cell line H1299. Neither of these mutants affects the binding of PTEN and AHCYL1 with PREX2 (Figure S7). In previous studies, the phosphorylation of AHCYL1 was important for its function, and protein phosphatase PP1α specifically dephosphorylated Ser^68^ of AHCYL1 35, 36. Once we co-expressed protein phosphatase PP1α with AHCYL1 and PREX2 in HEK293T cells, the interaction between PREX2 and AHCYL1 was abolished (Figure 3H). The phosphatase dead form of PP1α (R96A) and PP1α-pretreated with phosphatase inhibitors did not affect the interaction between PREX2 and AHCYL1 (Figure 3H and Figure S8). Similarly, when AHCYL1 was mutated to PP1α non-binding form (I42A/F44A, 2A), the interaction between AHCYL1 2A mutant and PREX2 was also not affected by PP1α (Figure 3H). Contrarily, AHCYL1 phospho-dead mutant (S68A) could not interact with PREX2 (Figure 3I). However, the phospho-mimic mutant (S68D) could not bind PREX2 either (Figure 3I), which suggested the subsequent phosphorylation of Ser^71^ and Ser^74^ induced by actual phosphorylation of Ser^68^ but not exist in the phospho-mimic mutant is also needed by PREX2-AHCYL1 interaction 35, 36. Since the region (1-104aa) that cascaded phosphorylation localizes did not interact with PREX2 (Figure 3E), the phosphorylation status of AHCYL1 probably conformationally affected the interface between PREX2 and AHCYL1 indirectly. These data proved that AHCYL1 was a direct binding partner of PREX2 and its phosphorylation status was critical for the interaction.

### AHCYL1 promotes NSCLC cell growth *in vitro* and* in vivo*

**Figure 4:**
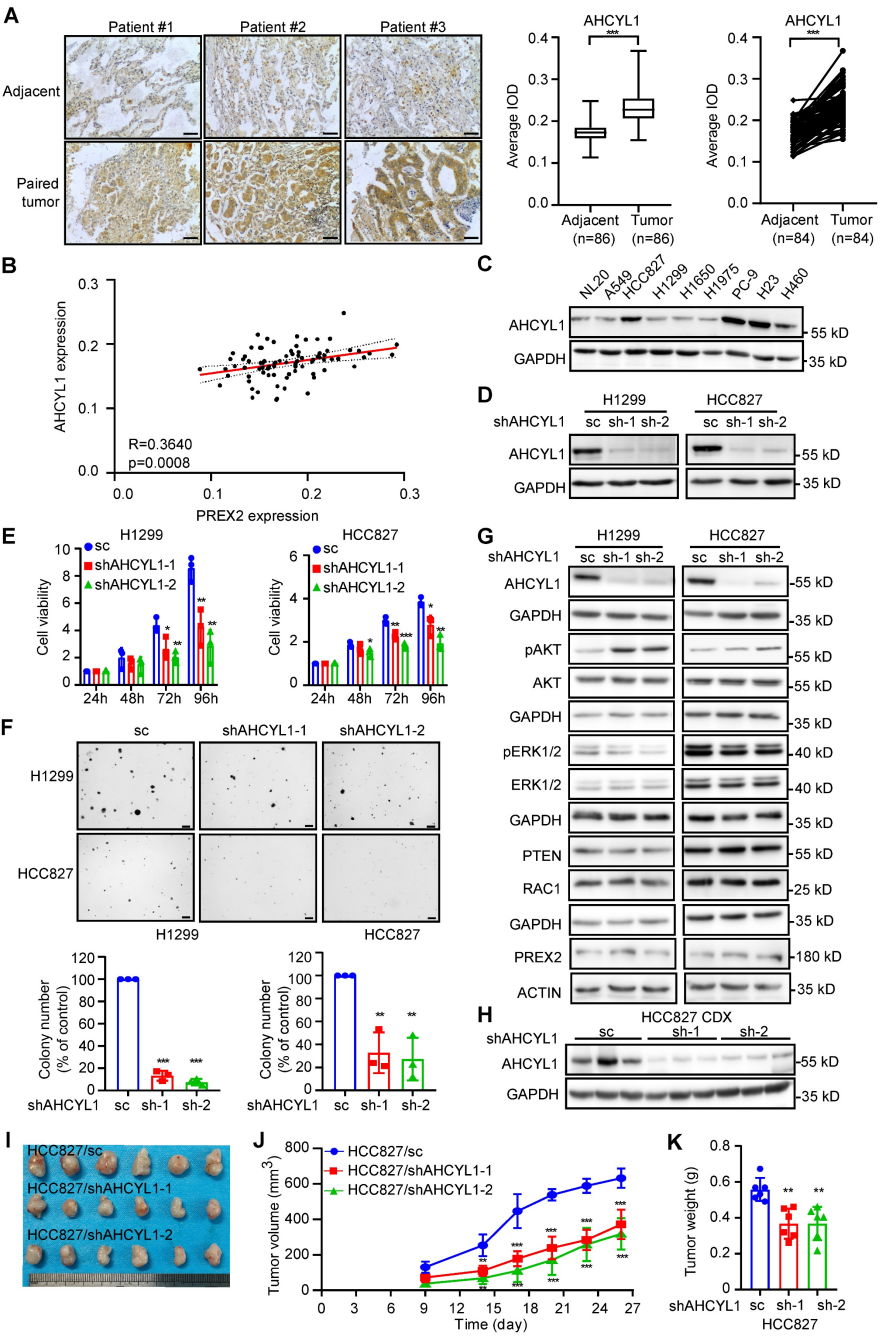
** AHCYL1 promotes cell growth of NSCLC cells *in vitro* and *in vivo*. A**. The expression of AHCYL1 was examined by immunohistochemistry (IHC) staining using a lung cancer tissue array. The left panels show representative images of paired lung cancer tissues and adjacent tissues. Scale bar:100 μm. The right panels show the quantification of all samples (adjacent tissues: n=86; tumor tissues: n=86) and paired samples (n=84). The statistical significance was determined by a two-tailed paired t test. ***, p < 0.001.** B.** The expression level of AHCYL1 and PREX2 in lung cancer tissue array showed a strong positive correlation (R=0.3640, p=0.0008). **C.** The expression of AHCYL1 in human normal lung epithelial cell NL20 and NSCLC cell lines was measured by western blotting. **D.** The level of AHCYL1 was measured in H1299 and HCC827 cells after knocking down by lentiviral transduction (sc: scrambled negative control of shRNA; shAHCYL1-1 and shAHCYL1-2). **E.** Cell viability of AHCYL1 knockdown NSCLC cells after cell seeding for 24, 48, 72 and 96 h was determined by MTT assay. Data are presented as means ± SD from 3 independent experiments (n = 3). Statistical significance was determined using multiple t-test. *, p < 0.05. **, p < 0.01. ***, p < 0.001. **F.** Anchorage-independent growth of NSCLC cells with AHCYL1 knockdown was determined by soft agar assay. Representative images are shown in the upper panels. Colonies with a dimeter more than 50 μm were counted using Image-Pro Plus (v.6) computer software and normalized with control group. Data are presented as means ± SD from 3 independent experiments. The statistical analysis was determined using one-way ANOVA. Scale bar: 200 μm. **, p < 0.01. ***, p < 0.001. **G.** The MEK/ERK and PI3K/AKT signaling pathways were detected by western blotting after AHCYL1 knockdown in H1299 and HCC827 cells. pAKT (Ser473) and pERK1/2 (Thr202/Tyr204) were measured. **H.** The level of AHCYL1 in the tumor tissues collected from control and AHCYL1 knockdown cells injected mice was detected by western blotting. **I.** The tumors collected from control and AHCYL1 knockdown cells injected mice are shown (n=6). **J.** The longitudinal tumor volume in the control and AHCYL1 knockdown mice group were measured and calculated every 3-4 days. Data are presented as means ± SD (n=6). Statistical significance was determined using one-way ANOVA. **, p < 0.01. ***, p < 0.001. **K.** The final tumor weights in the control and AHCYL1 knockdown mice groups were measured at the endpoints. Data are presented as means ± SD (n=6). Statistical significance was determined using one-way ANOVA. **, p < 0.01.

AHCYL1 (also called IRBIT) was originally identified as a binding protein of the intracellular Ca^2+^ channel inositol 1,4,5-trisphosphate (IP3) receptor and functions as an inhibitory regulator of this receptor 35, 37. Subsequently, multiple ion channels and ion transporters, such as the Na^+^/HCO_3_^-^ co-transporter NBCe1-B and NBCn1, the Na^+^/H^+^ exchanger NHE3, the Cl^-^ channel cystic fibrosis transmembrane conductance regulator (CFTR) and the Cl^-^/HCO_3_^-^ exchanger Slc26a6 and AE2 were identified as AHCYL1-binding partners 38-42. However, the pathological functions of AHCYL1 in NSCLC are still not quite clear. By IHC analysis with 90 paired human lung cancer and matched adjacent tissue arrays, we found that AHCYL1 expression level in tumor tissues was relatively higher than that in matched adjacent tissues (Figure 4A) and positively correlated with PREX2 expression (Figure 4B). However, there was no significant difference between tumors across ages, clinical stages and grades** (**Figure S9A). Kaplan-Meier analysis showed no difference in survival probability between patients with high and low levels of AHCYL1 (Figure S9B-D). We also evaluated AHCYL1 expression in NSCLC cell lines. Our results showed a higher expression level of AHCYL1 in 4 human NSCLC cell lines compared with normal lung epithelial cell NL20 (Figure 4C). We knocked down the expression of AHCYL1 in H1299 and HCC827 cells and performed cell-based functional assays to determine its contribution to the tumor phenotype (Figure 4D-F). Our results showed that the knockdown of AHCYL1 inhibited cell proliferation and anchorage-independent cell growth in NSCLC cells (Figure 4E-F). Moreover, we observed the increment of anchorage-independent cell growth after the overexpression of AHCYL1 (Figure S10A-B). We next checked PREX2-related signaling pathways and found that AHCYL1 positively regulated the phosphorylation of ERK1/2 but suppressed the phosphorylation of AKT (Figure 4G and Figure S10C). The knockdown of AHCYL1 in NL20 that has a similar AHCYL1 expression level compared with H1299 also suppressed cell growth (Figure S11A). However, the phosphorylation of ERK1/2 and AKT in NL20 was not affected (Figure S11B), which suggested that AHCYL1 might employ distinct regulatory mechanisms for cell growth in normal versus lung cancer cellular contexts. The results obtained from the cell-derived xenograft (CDX) model seeded with AHCYL1 knockdown HCC827 cells were consistent with *in vitro* findings. Tumor size and tumor weight were lower in the AHCYL1 knockdown group (Figure 4H-K). The phosphorylation of ERK1/2 and MEK1/2 was decreased, but the phosphorylation of AKT was increased in the AHCYL1 knockdown group (Figure S4B). These findings suggested that AHCYL1 promoted NSCLC cell growth *in vitro* and* in vivo* and regulated the PREX2-related signaling pathways.

### AHCYL1 enhances the GEF activity of PREX2 *in vitro* and *in vivo*

**Figure 5:**
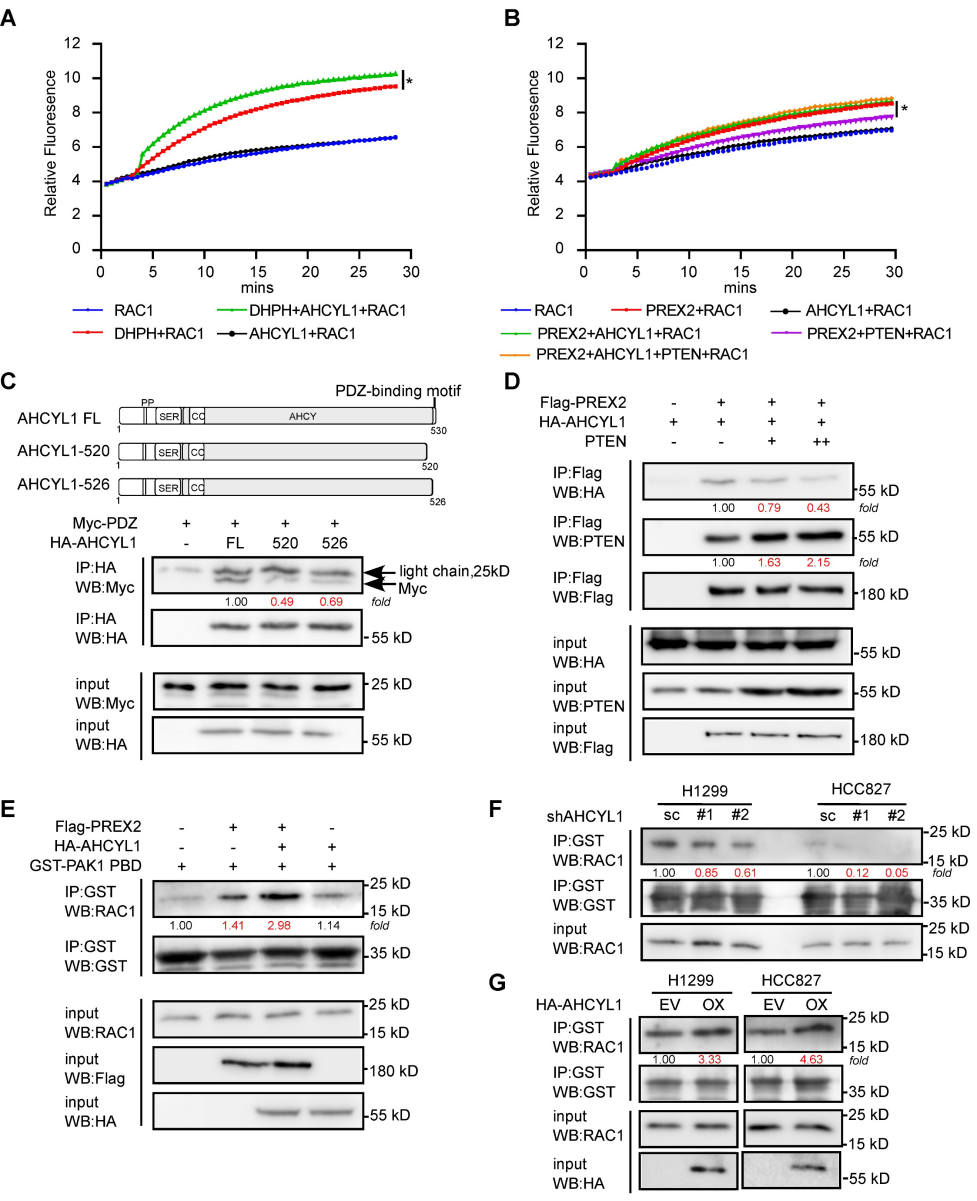
** AHCYL1 enhances the GEF activity of PREX2 *in vitro* and* in vivo*. A.** The effect of AHCYL1 on the GEF activity of PREX2 DH-PH domain was evaluated by *in vitro* GEF assay. Data are representative of triplicate experiments. Statistical significance was determined using one-way ANOVA. *, p < 0.05. **B.** The effect of AHCYL1 on the GEF activity of full-length PREX2 with or without PTEN was evaluated by *in vitro* GEF assay. Data are representative of triplicate experiments. Statistical significance was determined using one-way ANOVA. *, p < 0.05. **C.** Co-immunoprecipitation of Myc-PDZ with full-length AHCYL1 (FL) and C-terminal PDZ-binding motif truncations (520, 526) in HEK293T cells was performed with HA antibody and detected by Myc antibody. The full-length AHCYL1 and C-terminal PDZ-binding motif truncations are represented in graphical form. **D.** The competitive binding of AHCYL1 and PTEN with PREX2 was detected by anti-Flag immunoprecipitation in HEK293T cells. **E.** The GTP-bound active RAC1 in HEK293T cells transfected with Flag-PREX2 with or without HA-AHCYL1 were pulled down by GST-PAK1PBD and determined by western blotting. **F.** The GTP-bound active RAC1 in AHCYL1 knockdown H1299 and HCC827 cells were pulled down by GST-PAK1PBD and determined by western blotting. **G.** The GTP-bound active RAC1 in AHCYL1 overexpression H1299 and HCC827 cells were pulled down by GST-PAK1PBD and determined by western blotting. The intensity of the protein band was quantified with ImageJ software and normalized with control.

The data above illustrated the interaction pattern and regulatory potentials between PREX2 and AHCYL1. However, the underlined molecular mechanism remains unknown. Based on our co-IP assays, AHCYL1 interacts with the DH-PH domain of PREX2 which facility the GEF catalytic activity toward RAC1 (Figure 3F). So, we raised the question of whether AHCYL1 could affect the GEF activity of PREX2. By *in vitro* fluorescent GEF assay, the addition of AHCYL1 enhanced the GEF activity of the DH-PH domain toward RAC1 while AHCYL1 alone did not affect the exchange of GDP for GTP on RAC1, which suggested that AHCYL1 interacted with the DH-PH domain of PREX2 and directly enhanced its GEF activity *in vitro* (Figure 5A). Besides, the GEF assay showed that the purified full-length PREX2 could increase the exchange of GDP for GTP on RAC1 in a less extant than DH-PH domain; however, the addition of AHCYL1 alone did not dramatically affect the GEF activity of full-length PREX2 toward RAC1 (Figure 5B). But intriguingly, when AHCYL1 and PTEN coexist in the reaction, AHCYL1 could reverse the inhibition of PTEN on the GEF activity of full-length PREX2 (Figure 5B).

Furtherly, we tried to address the mechanism of how AHCYL1 releases the inhibition of PTEN. A previous study showed that the C-terminal tail of PTEN was structurally anchored to the PDZ2 domain of PREX2 and allosterically promoted an autoinhibitory conformation of PREX2 33. Our co-IP study showed that AHCYL1 could also bind with the PDZ2 domain of PREX2 (Figure 3F) and contains the C-terminal PDZ-binding motif tail. Deleting the C-terminal tail of AHCYL1 weakened its interaction with Myc-PDZ significantly compared with the full-length of AHCYL1 (FL, Figure 5C), which suggested that AHCYL1 binds the PDZ2 domain of PREX2 through its C-terminal tail similar as PTEN. Considering that AHCYL1 and PTEN both interacted with the PDZ2 domain of PREX2 by their C-terminal tails, we then investigated whether PTEN and AHCYL1 competitively bind PREX2. We co-transfected Flag-PREX2 and HA-AHCYL1 with the increasing amount of PTEN plasmid into HEK293T cells. Indeed, the results showed that increasing levels of PTEN expression reduced the binding between PREX2 and AHCYL1 (Figure 5D). These findings suggested that AHCYL1 interacted with the PDZ2 domain of PREX2 through its C-terminal PDZ-binding motif tail, thus abrogating PTEN inhibition and recovering the GEF activity of full-length PREX2. Because the inhibition between PREX2 and PTEN is mutual 17, the competitive binding of AHCYL1 could also increase the phosphatase activity of PTEN, which explains why AHCYL1 knockdown promoted the phosphorylation of AKT (Figure 4G).

To further confirm the effect of AHCYL1 on the GEF activity of PREX2 *in vivo*, we performed RAC activation pulldown assay in cells. The results showed that HEK293T cells that co-transfected with PREX2 and AHCYL1 possessed higher levels of activated RAC1 than that transfected with PREX2 alone (Figure 5E). In AHCYL1 knockdown NSCLC cells, the amount of activated RAC1 was decreased (Figure 5F). And NSCLC cells overexpressing AHCYL1 harbored higher levels of activated RAC1 (Figure 5G). Taken together, these results suggested that AHCYL1 could enhance the GEF activity of PREX2 *in vitro* and *in vivo*.

### AHCYL1 mediates the tumor-promoting effect of PREX2 in NSCLC cells

**Figure 6:**
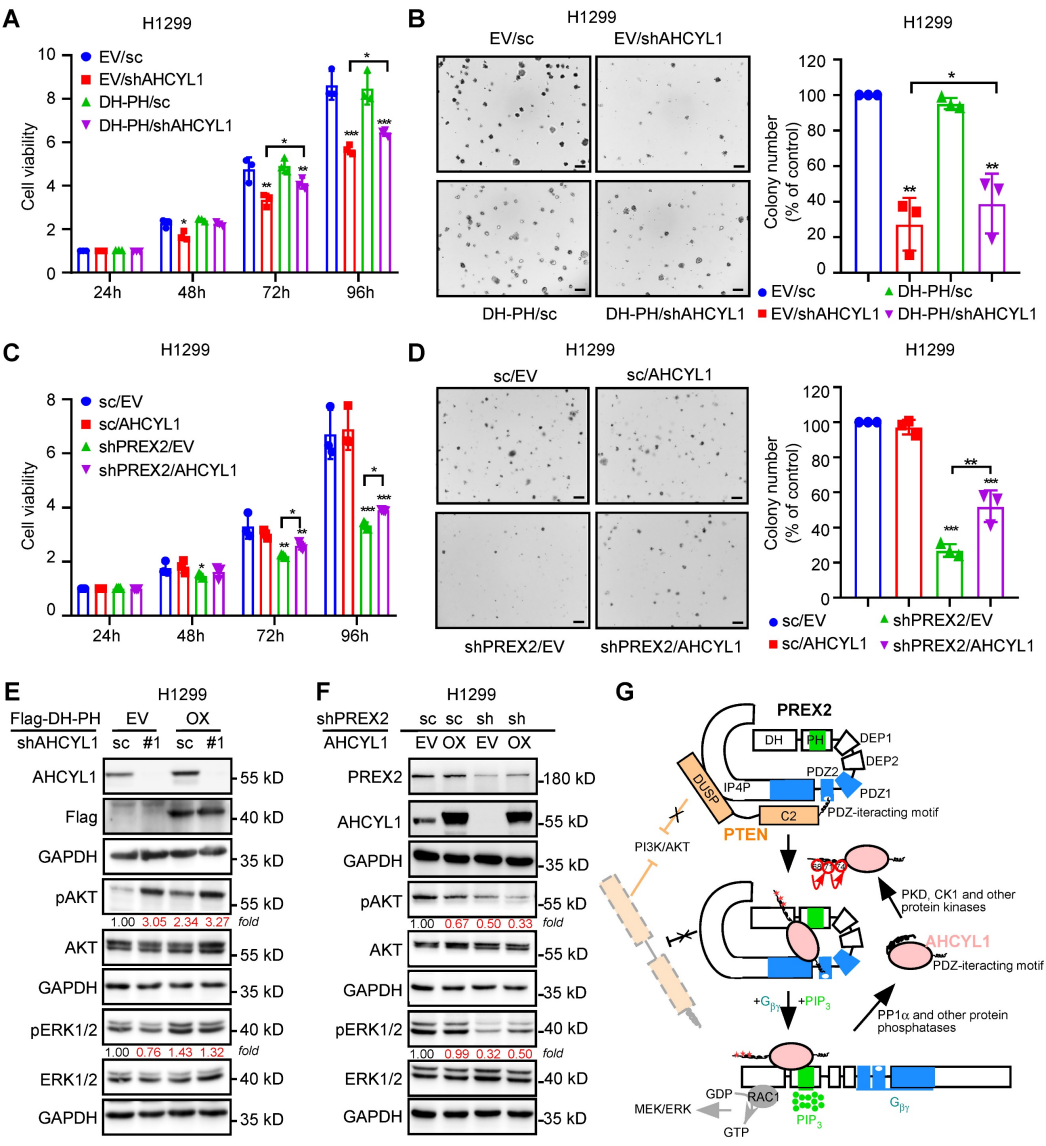
** AHCYL1 mediates the tumor-promoting effect of PREX2 in NSCLC cells. A.** Cell viability of H1299 cells with AHCYL1 knockdown upon PREX2 DH-PH domain overexpression was detected by MTT assay after cell seeding for 24, 48, 72 and 96 h. Data are presented as means ± SD from 3 independent experiments (n = 3). Statistical significance was determined using multiple t-test. *, p < 0.05. **, p < 0.01. ***, p < 0.001. **B.** Anchorage-independent growth of H1299 cells with AHCYL1 knockdown upon PREX2 DH-PH domain overexpression was detected by soft-agar assay. Colonies were counted using Image-Pro Plus (v.6) computer software. Scale bar: 200 μm. Data are presented as means ± SD from 3 independent experiments. The statistical analysis was determined using one-way ANOVA. Scale bar: 200 μm. *, p < 0.05. **, p < 0.001. **C.** Cell viability of H1299 cells with AHCYL1 overexpression after PREX2 silencing was assessed by MTT assay after cell seeding for 24, 48, 72 and 96 h. Data are presented as means ± SD from 3 independent experiments (n = 3). Statistical significance was determined using multiple t-test. *, p < 0.05. **, p < 0.01. ***, p < 0.001. **D.** Anchorage-independent growth of H1299 cells with AHCYL1 overexpression after PREX2 silencing was assessed by soft agar assay. Colonies were counted using Image J-Plus. Scale bar: 200 μm. Data are presented as means ± SD from 3 independent experiments. The statistical analysis was determined using one-way ANOVA. Scale bar: 200 μm. **, p < 0.01. ***, p < 0.001. **E** and **F**. ERK/MEK and PI3K/AKT signaling pathways were detected by western blotting in H1299 cells with AHCYL1 knockdown upon PREX2 DH-PH overexpression (**E**) and AHCYL1 overexpression after PREX2 silencing (**F**). The intensity of the protein band was quantified with ImageJ software and normalized with control. **G.** Schematic illustration of our model for the interaction and regulation of the PREX2 and AHCYL1 complex. The mutual inhibition between PREX2 and PTEN is released by the competitive binding of phosphorylated AHCYL1 through its C-terminal tail. The direct interaction between AHCYL1 and the DH-PH domain of PREX2 further enhances the GEF activity of PREX2. The interaction between PREX2 and AHCYL1 is abolished when protein phosphatase PP1α dephosphorylates AHCYL1. The cascaded phosphorylation of AHCYL1 at Ser^68^, Ser^71^ and Ser^74^ was labeled with red asterisks.

To further verify the regulation of AHCYL1 on PREX2, we manipulated the expression of AHCYL1 upon DH-PH domain overexpression in NSCLC cells, H1299. The results showed that AHCYL1 knockdown suppressed the cell proliferation and anchorage-independent cell growth of the control cells and DH-PH domain overexpression cells (Figure 6A-B). But, due to the tumor-promoting effect of DH-PH overexpression, the growth inhibitory effect caused by AHCYL1 knockdown in overexpressing DH-PH cells was less than that observed in cells transfected with the empty vector (Figure 6A-B).

Next, we overexpressed AHCYL1 in PREX2 knockdown H1299 cells. The cell-based functional assays showed that AHCYL1 overexpression partially reversed the growth inhibitory effect caused by PREX2 knockdown (Figure 6C-D). Correspondingly, in AHCYL1 knockdown upon DH-PH domain overexpression cells, the higher phosphorylation level of ERK1/2 induced by DH-PH domain overexpression was reduced by AHCYL1 knockdown (Figure 6E). However, with less AHCYL1 competitive binding, PREX2 suppressed the phosphatase activity of PTEN and the phosphorylation of AKT was boosted (Figure 6E). In PREX2-knockdown cells, AHCYL1 overexpression partially recovered the ERK1/2 phosphorylation and furtherly suppressed the AKT signaling because of the releasing of mutual inhibition between PREX2 and PTEN (Figure 6F). The overexpression of PREX2 non-binding mutant AHCYL1 S68A could not rescue the cell growth inhibition caused by PREX2 knockdown and did not affect the AKT and ERK1/2 phosphorylation in PREX2-knockdown cells (Figure S12). Taken together, these results demonstrate that AHCYL1 mediates the tumor-promoting effect of PREX2 by regulating the binding and mutual inhibition between PREX2 and PTEN in NSCLC cells. Based on the binding pattern and the effects of AHCYL1 on the GEF activity of PREX2, we propose the following model to explain the regulation of AHCYL1 on PREX2 (Figure 6G). In the basal resting state, PREX2 is autoinhibited by its interdomain interaction between the DH-PH and IP4P domains. PTEN binds to PREX2 by bridging the PREX2 PDZ2 domain and IP4P domains, which tightly locks PREX2 in the autoinhibited state, preventing its activation by G_βγ_ 33. The presence of phosphorylated AHCYL1 competes with PTEN for interaction with the PDZ2 domain of PREX2, thus releasing the PTEN inhibition on PREX2. Once the binding of G_βγ_ and PIP3 confers PREX2 to an open conformation, the direct interaction between AHCYL1 and PREX2 DH-PH domain further causes an additional enhancement of the PREX2 GEF activity. The interaction between AHCYL1 and PREX2 is abolished when AHCYL1 is dephosphorylated by PP1α. In this model, the mechanism whereby the phosphorylation and dephosphorylation of AHCYL1 confer the interaction and AHCYL1 directly regulates PREX2 DH-PH catalytic activity still are unclear.

## Discussion

Lung cancer remains the leading cause of cancer-related mortality globally, necessitating continuous exploration of novel therapeutic targets and mechanisms underlying lung cancer progression. Our study highlights the oncogenic roles of PREX2 as GEF factor and PTEN suppressor in NSCLC, offering insights into its potential as a therapeutic target. GEFs are not generally considered good targets for pharmacological inhibition because of the relatively large surface area through which they interact with their target GTPase 43. However, several small-molecule inhibitors that target the interface between PREX1/PREX2 and Rac, such as NSC23766 and PREX2-in 1, have been developed and shown preclinical efficacy in other cancers 44-46. But it seems likely that long-term treatment with these inhibitors induced cytotoxicity caused by off-target effect. And it remains to be investigated further before these compounds can be used in animal models. Our data identified AHCYL1's role as a PREX2 activator. Developing compounds using peptide mimetics to block the AHCYL1-PREX2 interface may indirectly attenuate RAC1 signaling in NSCLC. Our data demonstrate that phosphorylation of AHCYL1 at Ser⁶⁸ is critical for its interaction with PREX2. While the exact structural consequences of this phosphorylation remain to be fully elucidated, existing studies suggest that post-translational modifications, such as phosphorylation, often induce conformational changes in proteins by altering charge distribution or exposing binding interfaces. For instance, phosphorylation of IRBIT (AHCYL1) at specific residues regulates its ability to bind IP3 receptors by modulating its tertiary structure and affinity for partner proteins 35. Future structural studies, such as cryo-EM or X-ray crystallography of phosphorylated versus non-phosphorylated AHCYL1, could clarify these mechanistic details. Additionally, phosphorylation-dependent interactions are often regulated by upstream kinases or phosphatases. Inhibiting the kinase responsible for Ser⁶⁸ phosphorylation of AHCYL1 to eliminate AHCYL1-PREX2 interaction may be another strategy of targeting PREX2. The combination of inhibitors targeting PREX2-RAC1 directly and indirectly may overcome the cytotoxicity and specificity problem of known GEF inhibitors. Our data supported the potential of PREX2 and AHCYL1 as the therapeutic targets of NSCLC. However, the bioinformatic analysis with the TCGA cohort only and another larger cohort integrated by Kaplan-Meier Plotter showed differential impact of PREX2 on the prognosis of distinct NSCLC patient subgroups (Figure S1). The different databases include patients of different ethnicities, ages, and clinical stages. The heterogeneity of lung cancer tumors and the variations in analytical methods may result in these seemingly differential results, which reflect the complexity of cancer biology and clinical research, requiring deeper investigation and further validation. The combined effect of PREX2 and AHCYL1 on the prognosis of NSCLC patients also showed no differences between the patients with high PREX2+high AHCYL1, high PREX2+low AHCYL1, low PREX2+high AHCYL1 and low PREX2+low AHCYL1 (data not shown). Therefore, the prognostic value of PREX2 and AHCYL1 need further refined investigation and may need other known prognostic markers to support them.

Notably, AHCYL1 is a multifunctional protein with established roles in ion homeostasis, including regulation of IP3 receptors and other plasma membrane transporters 35, 38-40. AHCYL1's dual roles in ion homeostasis and tumor promotion warrant caution; systemic inhibition could perturb normal cellular physiology. However, the tumor-specific reliance on the PREX2-AHCYL1 axis, as evidenced by different MEK/ERK and PI3K/AKT signaling response in NL20 and NSCLC cells after AHCYL1 knockdown, suggests a potential therapeutic window. And another key question is whether the interaction with PREX2 is required for canonical functions of AHCYL1. The tumor-promoting effects of AHCYL1 in NSCLC appear to rely heavily on its partnership with PREX2, as AHCYL1 knockdown specifically disrupts PREX2 GEF related signaling pathways in NSCLC but not in normal lung epithelia cell NL20 with low expression of PREX2. This dichotomy underscores the context-dependent nature of AHCYL1's functions: while its ion transport roles may operate independently, its oncogenic activity in NSCLC is tightly linked to PREX2. Further studies using AHCYL1 mutants defective in PREX2 binding but retaining ion channel regulatory capacity and the detection of intracellular Ca²⁺ flux in PREX2-knockdown cells could dissect these pathways.

In our study, the cell growth inhibition phenotype of AHCYL1 knockdown and the enhancement on the GEF activity of PREX2 suggest AHCYL1 possesses the tumor-promoting effect. At the same time, AHCYL1 releases the mutual inhibition between PREX2 and PTEN, thus leading PTEN facility its tumor suppressor function. In other studies, under the stimulation with epidermal growth factor (EGF), AHCYL1 expression is enhanced to maintain the expression of the NBCn1 transporter machinery in the plasma membrane, which plays a positive role in the migration of lung cancer cells 47. Meanwhile, AHCYL1 can also regulate cell plasticity to inhibit lung cancer tumorigenesis 48. Therefore, the role of AHCYL1 in NSCLC tumor progression is paradoxical. In other cancers, the role of AHCYL1 in tumor progression is also distinguished. In human ovarian epithelial cancer, AHCYL1 expression is decreased and high expression of AHCYL1 is a favorable factor for overall responses and progression-free survival 49. These observations, associated with the fact that AHCYL1 expression was found to be reduced in human malignant melanoma cell lines that are resistant to DNA-damaging drugs 50, highlight the key role of AHCYL1 as a tumor suppressor. But in some cholangiocarcinoma patients, AHCYL1 presented tumor-promoting capacity through fusing with fibroblast growth factor receptor 2 (FGFR2) to form a chimeric protein which shows constitutive tyrosine phosphorylation in the activation loop of the FGFR2 kinase domain and the hyperactivation of MAPK 51. Thus, it may be not appropriate to define AHCYL1 simply as an oncogene or a tumor suppressor remote from its specific regulatory mechanism. Resolving the paradoxical roles of AHCYL1—as both a tumor suppressor and promoter—requires deeper mechanistic dissection. For instance, AHCYL1's phosphorylation status or subcellular localization may dictate its functional output.

In conclusion, our study establishes PREX2 and AHCYL1 as critical drivers of NSCLC progression and unveils a novel regulatory mechanism linking AHCYL1 to PREX2 activation. These findings not only advance our understanding of NSCLC pathogenesis but also highlight actionable targets for therapeutic intervention. Future efforts should focus on translating these insights into targeted strategies, leveraging structural biology and drug discovery to combat this devastating disease.

## Materials and Methods

### Plasmid construction

The PREX2-V5/His (Cat#41555, Addgene, Cambridge, MA) was a gift from Ramon Parsons. The expression vector HA-AHCYL1 (Cat#HG16909-NY) on pCMV3-N-HA was purchased from SinoBiological (Beijing, China). The expression vector Flag-PP1α (Cat#G109841) on pcDNA3.1 was purchased from Youbio Biotechnology Company (Changsha, China). Other expression constructions were made from their corresponding original plasmid with the primers listed in the Table S1. And the details for constructions were represented in supplementary materials and methods. For the shRNA plasmids, shPREX2-1 (5'-CGAATTTGTGTCATGGCTGTT-3'), shPREX2-2 (5'-GAACAGGGTGAGAAACTTTAT-3'), shAHCYL1-1 (5'-CGGCAAGTCGATGTCGTAATA-3') and shAHCYL1-2 (5'-CAATGTCTAAATCGCCTTAAA-3') were cloned into the pLKO.1 backbone (Addgene plasmid # 8453). The psPAX2 (a packaging vector) and pMD2.G (an envelope vector) were purchased from Addgene (plasmid #12259 and #12260). The pLKO.1-puro non-target shRNA Control Plasmid DNA (scramble, sc) was purchased from Sigma-Aldrich.

### Cell culture

The human normal lung epithelial cell (NL-20) and all lung cancer cell lines were purchased from American Type Culture Collection (ATCC). The cells were routinely screened to confirm Mycoplasma-negative status and to verify the identity of the cells by short tandem repeat profiling before being frozen. Each vial was thawed and maintained for a maximum of 2 months. Cells were cultured at 37 °C in a 5% CO_2_ humidified incubator following the ATCC protocols. Normal lung cell NL-20 was cultured in Ham's F12 medium with 1.5 g/L sodium bicarbonate, 2.7 g/L glucose, 2.0 mmol/L L-glutamine, 0.1 mmol/L nonessential amino acids, 0.005 mg/ml insulin, 10 ng/mL epidermal growth factor, 0.001 mg/mL transferrin, 500 ng/mL hydrocortisone and 4% fetal bovine serum. Human lung cancer cells were all cultured with RPMI-1640 medium containing 10% FBS, 1% penicillin and streptomycin (Gen DEPOT, Katy, TX, USA). HEK293T cells (stably expressing the SV40 large T antigen in HEK293 cells) were purchased from the ATCC and cultured in DMEM medium supplemented with 10% FBS, 1% penicillin and streptomycin.

### Lentivirus production and infection

To generate PREX2 or AHCYL1 knockdown cells, the lentiviral shRNA plasmids or control plasmid DNA (scramble, sc) were transfected into HEK293T cells together with the packaging vector psPAX2 and envelope vector pMD2.G using jetPRIME transfection reagent (Cat#101000046, Polyplus) following the manufacturer's suggested protocols. The transfection mixture was incubated with cells for 12 h, and then 10 mL of fresh DMEM medium with 10% FBS and antibiotics (penicillin/streptomycin) were added. Viral supernatant fractions were collected at 48 h and 72 h after transfection. The pooled supernatant fractions were then filtered through a 0.45 µm filter and frozen at -80 °C for later use. The appropriate cells were infected with the viral supernatant fraction together with 8 µg/ml polybrene (Cat#TR-1003-G, Sigma-Aldrich, St. Louis, MO, USA). After overnight infection, cells were subcultured with fresh complete growth medium containing the appropriate concentration of puromycin. For H1299, 8 µg/ml puromycin was added. For HCC827, 2.5 µg/ml puromycin was added. The cells were selected with puromycin for 48 h to 72 h and used for further analysis.

### Immunohistochemistry staining

The 90 paired human lung cancer tissue and matched adjacent tissue array was obtained from the Tufeibio company (Cat#TFLungade-01, Shanghai, China). The array was deparaffinized in xylene solution and rehydrated using gradient ethanol concentrations. Antigen retrieval was performed using sodium citrate and the slides were then incubated with H_2_O_2_ to block endogenous peroxidases. Thereafter, the primary antibodies, PREX2 (Cat#ab121462, 1:100) or AHCYL1 (Cat#H00010768-M05, 1:100), were incubated at 4 °C overnight and the signals were visualized by the indirect avidin biotin-enhanced horseradish peroxidase method according to the manufacturer's instructions (Vector Laboratories, Burlingame, CA). After mounting, all sections were observed by microscope and quantitative analysis was performed by calculating the average integrated optical density (IOD) value measured by Image J software.

### Western blotting analysis

Cells were rinsed with ice-cold phosphate-buffered saline (PBS) and disrupted in RIPA lysis buffer (Cat#R0020, Solarbio) or IP lysis buffer containing 50 mM Tris-HCl (pH 7.4), 150 mM NaCl, 1 mM EDTA, 10% (v/v) Glycerol, 0.5% (v/v) NP40 and protease inhibitor cocktail. The cell lysate was incubated on ice for 15 min and centrifuged at 12000 rpm for 15 min at 4 °C. The protein concentration of the cleared cell lysates was determined using the bicinchoninic acid (BCA) Protein Assay Kit (Cat#PC0020, Solarbio), following the manufacturer's instructions. Cell lysates were separated by SDS-PAGE and transferred to Immobilion-PVDF membrane (Cat#IPVH00010, Millipore) in transfer buffer containing 20 mM Tris-HCl (pH 8.0), 150 mM glycine and 20% (v/v) methanol. Membranes were blocked with 5% non-fat milk in Tris-buffered saline containing 0.05% Tween-20 (TBST) at room temperature for 1 h and incubated with specific primary antibodies at 4 °C overnight. The membranes were washed three times using TBST, and appropriate horseradish peroxidase-conjugated secondary antibody incubation was performed at room temperature for 1-2 h. The membranes were then incubated with the enhanced chemiluminescence reagent (Cat#MA0186, Meilunbio, Dalian, China), and the target bands were visualized using the Amersham Imager 600 (GE Healthcare Life Science, Pittsburgh, PA, SA).

### Cell assays: MTT assay and soft agar assay

For MTT assay, 3 × 10^3^ of cells were seeded in 96-well plates. After 24, 48, 72 and 96 h of culturing, the cells were incubated with 20 μL of 5 mg/mL MTT for 1.5 h. Afterward, the medium was discarded and replaced with 150 μL of DMSO. The absorbance at 490 nm was measured using the Multiskan GO Microplate Spectrophotometer (Thermo Scientific, Vantaa, Finland). All MTT experiment were repeated at least three times. Data are presented as means ± SD from 3 independent experiments (n=3).

For soft agar assay, RPMI-1640 medium supplemented with 10% FBS, 1% gentamicin, L-glutamine, were mixed with 0.5% agar, added 3 mL/well into 6-well plate and allowed to solidify over 2 h. 8 × 10^3^ of cells suspended in complete growth medium were mixed with 0.3% agar and added to the solidified bottom layer. Each cell lines were plated in 3 wells and incubated at 37 °C in a humidified atmosphere with 5% CO_2_ for 2-3 weeks. Afterwards, the colonies were photographed randomly from 5 areas of each well using the Olympus microscopic imaging system and the colonies with a diameter more than 50 μm were counted using the Image-Pro Plus software (v.6.0) (Media Cybernetics, Rockville, MD). All soft agar experiments were repeated at least three times and results are presented as mean ± SD from 3 independent experiments (n=3).

### Cell-derived xenograft mouse model

For PREX2 function, six-to-eight-week-old NU/NU mice (SPF Biotechnology Co., Ltd, Beijing, China) were randomly divided into three groups as follows: scramble (n=6); shPREX2-1 (n=6) and shPREX2-2 (n=6). And for AHCYL1 function, the mice groups are scramble (n=6), shAHCYL1-1 (n=6) and shAHCYL1-2 (n=6). 1 × 10^7^ of HCC827 cells infected with the indicated lentivirus were injected subcutaneously into the mice. Tumor volumes were measured every 3-4 days using a Vernier caliper and calculated as V = (length) × (width) × (height) × 0.52. Mice were euthanized and tumors were extracted when tumor volume reached 1000 mm^3^.

### *Prex2* knockout mice and urethane treatment

The conventional whole body* Prex2* knockout mice (*Prex2*-KO) were generated by CRISPR technology at the C57BL/6N genetic background in the Cyagen Biosciences, Inc. (Suzhou, China) and housed and bred under virus- and antigen-free conditions. Mice were genotyped by standard PCR analysis according to the genotyping protocol with specific primers Table S1). Mice (6-8 weeks old) were randomly divided into four groups: (1) WT-vehicle (8 female and 8 male), (2) *Prex2*-KO-vehicle (8 female and 8 male), (3) WT-Urethane (28 female and 25 male), and (4) *Prex2*-KO-Urethane (17 female and 20 male). The urethane-treated groups were subjected to a single i.p. injection of urethane (1 g/kg in 1 × PBS, Sigma) or vehicle (1 × PBS) once a week for 10 weeks. Mice were monitored every day and weighed once a week. Mice were euthanized by CO_2_ asphyxiation at 40 weeks after the first injection of urethane or when moribund. Tumors macroscopically visible on the pleural surface of the lungs were counted, and lungs were photographed and fixed in 4% formalin for histological analysis. All animal studies were performed according to guidelines approved by the Ethics Committee of Zhengzhou University (Zhengzhou, Henan, China). The assigned approval number is CUHCI2020035.

### GST pull-down

25 µL of AHCYL1-binding Glutathione magnetic beads and 250 ng of purified Flag-PREX2 protein were incubated on ice for 1 h with occasional mixing. The beads were washed three times with washing buffer (50 mM Tris-HCl pH 7.4, 300 mM NaCl, 0.5% NP40). Then, 30 µL of 2×loading buffer was added to the beads and supernatant samples. The samples were boiled at 95 °C for 10 min and resolved by SDS-PAGE.

### Immunoprecipitation

Cell pellets were incubated with IP lysis buffer containing 50 mM Tris-HCl, pH 7.4, 150 mM NaCl, 1 mM EDTA, 10% (v/v) Glycerol, 0.5% (v/v) NP40 and protease inhibitor cocktail for 30 min on ice and centrifuged at 12000 rpm for 15 min at 4 °C. The protein concentration of the cleared cell lysates was determined using the bicinchoninic acid (BCA) Protein Assay Kit (Cat#PC0020, Solarbio), following the manufacturer's instructions. After quantification, 1-2 mg appropriate cell lysates were incubated with specific antibodies and 25 μL of Protein A/G Magnetic Beads (Cat#HY-K0202, MCE) rotating for 4 h at 4 °C. The beads were washed four times with washing buffer, and the immune complexes were eluted at 95 °C for 5 min with 6×loading buffer. The immunoprecipitated complexes were then separated by SDS/PAGE and subjected to western blot analysis.

### *In vitro* GEF assay

The guanine nucleotide exchange (GEF) activity of PREX2 and PREX2 DH-PH domain were monitored using a RhoGEF Exchange Assay Biochem Kit (Cat#BK100, Cytoskeleton) according to the manufacturer's protocol. Recombinant proteins were expressed and purified as described in Supplementary materials and methods. The reactions were conducted in a 96-well black flat bottom half area plate (Cat# 3686, Corning, NY). Each reaction contains 1.5 µM RAC1 GTPases (provided in the kit), 0.75 µM N-MAR-GTP, 0.5 µM PREX2 or PREX2 DH-PH protein with or without the presence of 0.5 µM GST-AHCYL1 and 0.5 µM Flag-PTEN. Reactions were measured in a Tecan Spectrofluor plus fluorimeter (λ _ex_= 485 nm, λ _em_= 535 nm). Readings were taken every 30 s for a total reaction time of 30 min. Three independent assays were performed.

### Active RAC1 pull down assay

Active RAC1 pull down assay was conducted with Active Rac1 pull-down and detection kit (Cat#16118, ThermoFisher Scientific). HEK293T cells were transfected with plasmids as indicated for 36 h. After depletion of serum overnight, the cells were harvested and suspended in lysis buffer provided by kit. 2 mg of protein extracts were then incubated with 20 µg of GST-Pak1-PBD and 20 µL of Glutathione Resin at 4 °C for 1 h with mild agitation. The beads were then washed with lysis buffer for three times and resuspended in 30 µL of 2 × reducing loading buffer. The eluted samples were separated by 15% SDS/PAGE gel and subjected to Western blot analysis.

### Statistical analysis

All in vitro experiments were repeated at least three times and data are presented as mean ± SD unless otherwise noted. The number of replicates for each experiment (number of patients, number of independent experiments for *in vitro* cell-based assays, number of mice for *in vivo* animal studies) is indicated in the figure legends. Statistical analyses were conducted with GraphPad Prism 7 software. For MTT assay, multiple t-test were used to determine the statistical significance. For other assays, statistical significance was determined by Student's t-tests (comparing 2 groups) or one-way ANOVA (comparing three or more groups). A p-value of 0.05 was required to ascertain statistical significance. In this paper, the following conventions are used: *, p < 0.05; **, p < 0.01; and ***, p < 0.001.

## Supplementary Material

Supplementary materials and methods, figures and tables.

## Abbreviations

AHCYL1: adenosylhomocysteinase like 1
CDX: cell xenograft mouse model
GEF: guanine nucleotide exchange factor
HE: hematoxylin-eosin staining
IHC: immunohistochemistry
LC-MS/MS: liquid chromatography with tandem mass spectrometry
LUAD: lung adenocarcinoma
LUSC: lung squamous cell carcinoma
NSCLC: non-small cell lung cancer
PREX2: phosphatidylinositol-3,4,5-trisphosphate-dependent Rac exchanger factor 2
PDX: patient-derived xenograft mouse model
