# Supplementary material for: AHCYL1 mediates the tumor-promoting effect of PREX2 in non-small cell lung carcinoma
Source: Theranostics. 2025 Apr 21;15(12):5772–89. doi: 10.7150/thno.108654 (PMC12068309; doi:10.7150/thno.108654)
Supplement: Supplementary file 1 — Supplementary materials and methods, figures and tables. [file thnov15p5772s1.pdf]

Supplementary Materials for

AHCYL1 mediates the tumor-promoting effect of PREX2 in non-small cell lung carcinoma

Mingjuan Lei,[1,2] Yiu To Yeung,[2] Wenna Nie,[2] Ran Yang,[2] Jian Li,[2] Hanyong Chen,[3] Ran Zhao,[1,2] Kangdong Liu,[1,2,*] and Zigang Dong[1,2, *]

Correspondence to: dongzg@zzu.edu.cn (ZGD); kdliu@zzu.edu.cn (KDL)

This PDF file includes:

Materials and Methods
Figure. S1 to S12
Tables. S1
Tables. S2

Other Supplementary Materials for this manuscript include the following:

Materials and Methods

Plasmid construction

The PREX2-V5/His was a gift from Ramon Parsons (Cat#41555, Addgene). Using it as a DNA template, the full length and DH-PH domain of PREX2 were then PCR-amplified, N-terminal fused with flag-tag and cloned into the BamHI/XhoI sites of the pcDNA3.1 V5/His vector to construct the expression vector Flag-PREX2 and Flag-DHPH. To construct the Myc-tagged expression vector of the PREX2 domains, the DNA sequences corresponding to DH (aa 23–214), PH (aa 245–365), DEP1 (aa 390–465), DEP2 (aa 489–566), PDZ1 (aa 591–673) and PDZ2 (aa 675–758) were amplified by PCR using specific primers and PREX2-V5/His as a template. The PCR products were then digested and inserted into the XhoI/NotI sites of the pCMV-Myc vector.

The expression vector HA-AHCYL1 (Cat#HG16909-NY) on pCMV3-N-HA was purchased from SinoBiological. To construct the various AHCYL1 truncations, the corresponding DNA sequences were amplified by PCR using specific primers and HA-AHCYL1 as a template. The PCR fragments were cloned back into the KpnI/XbaI sites of the pCMV3-N-HA vector. The site-directed mutations of Ser68A (S68A) and I42A/F44A (2A) were performed using the QuickChange lightning site-directed mutagenesis kit (Agilent, Cat#210518) according to the manufacturer's instructions on HA-AHCYL1 vector. The AHCYL1 were transferred via PCR to pGEX6p-1, enabling purification of GST–AHCYL1.

The expression vector Flag-PP1α (Cat#G109841) on pcDNA3.1 was purchased from Youbio Biotechnology Company (Changsha, China). The site-directed mutation of R96A was performed using the QuikChange lightning site-directed mutagenesis kit (Agilent, Cat#210518) according to the manufacturer's instructions.

For the overexpression plasmids, the Flag-tagged DH-PH domain of PREX2 fragment and HA-tagged AHCYL1 were cloned into XbaI/NotI sites of the pCDH-CMV-MCS-PURO backbone. All constructs were confirmed by restriction enzyme mapping, DNA sequencing and the Blast program. The sequences of all primers used for plasmid construction are also listed in Supplementary Table 1.

Pull down assay and LC-MS/MS

For pulldown of PREX2-interacting protein, 50 µl of PREX2-binding V5 bead slurry was incubated with A549, H1299 and HCC827 cell lysates separately for 6 h rotating at 4°C. The beads were washed five times in washing buffer and resuspended in 20 µl of 2× loading buffer. The samples were then analyzed by SDS-PAGE and the bands were excised. The protein bands were digested overnight with sequencing grade trypsin. These digests were analyzed by microcapillary liquid chromatography/tandem mass spectrometry (LC-MS/MS) and the MS/MS spectra were searched against the reversed and concatenated Swiss-Prot protein database.

Purification of recombinant proteins

For Flag-PREX2, Flag-DHPH and Flag-PTEN protein purification, HEK293T cells were transfected with the expression vectors separately for 48h. The cell pellets were resuspended in

IP lysis buffer, sonicated and centrifuged at 12,000rpm for 15 mins at 4°C. 200 µl of 50% (v/v) anti-Flag® M2 affinity gel (Cat#A2220, Millipore) were added to cell lysate. The mixtures were incubated and rotated at 4°C for at least 4 hours to enable sufficient binding of the flag-fusion proteins to the beads. The fusion protein-binding beads were then washed with washing buffer (50 mM Tris, pH 7.4, 300 mM NaCl, 0.1% (v/v) NP40) for five times. Finally, the flag-fusion proteins were eluted by 0.2 mg/ml Flag peptides (Cat#F3290, Millipore) dissolved in elution buffer (25 mM Tris, pH 7.4, 150 mM NaCl). The purity of recombinant proteins was determined by SDS-PAGE followed by Coomassie Blue staining. For V5-PREX2 protein purification, the methods were the same as above, except using 0.2 ml of 50% (v/v) Protein A/G agarose bead slurry and 20 µl of V5 antibody to bind with V5-PREX2 protein.

For GST-fusion protein, BL21 (DE3) pLysS E. coli strain was transformed with the pGEX-6p-1/AHCYL1plasmid. Single colony was cultured in LB medium (supplemented with 100 µg / ml ampicillin) at 37 °C with vigorous shaking overnight. 3 ml bacteria were transferred into 300 ml fresh LB medium and cultured to an optical density at 600 nm (OD600) of 0.6-0.8. Then, 0.2 mM isopropyl β-d-1-thiogalactopyranoside (IPTG, Cat#I5502, Sigma-Aldrich) was added to the medium and cultures were continued to induced at 16 °C overnight. Cells were collected by centrifugation at 5,000g at 4 °C for 10 min. Extracts of cells were prepared by sonication in bacteria lysis buffer (50 mM Tris, 150 mM NaCl, 1 mM EDTA, 10% glycerol, 0.5% NP-40, 2 mM DTT, Protease Inhibitor Cocktail). Lysates were clarified by centrifugation at 12,000 rpm at 4 °C for 15 min, and the resulting supernatant was incubated with 300 µl of PierceTM magnetic agarose (Cat#78602, ThermoFisher Scientific) for 2 h at 4 °C. The beads were washed once with 10 ml of high salt washing buffer (50 mM Tris, 500 mM NaCl, 0.5% NP-40, 1 mM DTT) followed by 10 ml of low salt washing buffer (50 mM Tris, 300 mM NaCl, 0.5% NP-40, 1 mM DTT) for three times. GST-tagged AHCYL1 was eluted eight times with 500 µl of elution buffer (50 mM Tris, 150 mM NaCl, 10 mM glutathione, 1 mM DTT). Fractions containing GST-AHCYL1 were pooled together, concentrated and reconstituted in protein buffer (25 mM Tris, 150 mM NaCl, 2 mM DTT) in an Amicon Ultra-4 Centrifugal Filter Unit (UFC801024, Sigma-Aldrich). The samples were stored at −80 °C until use.

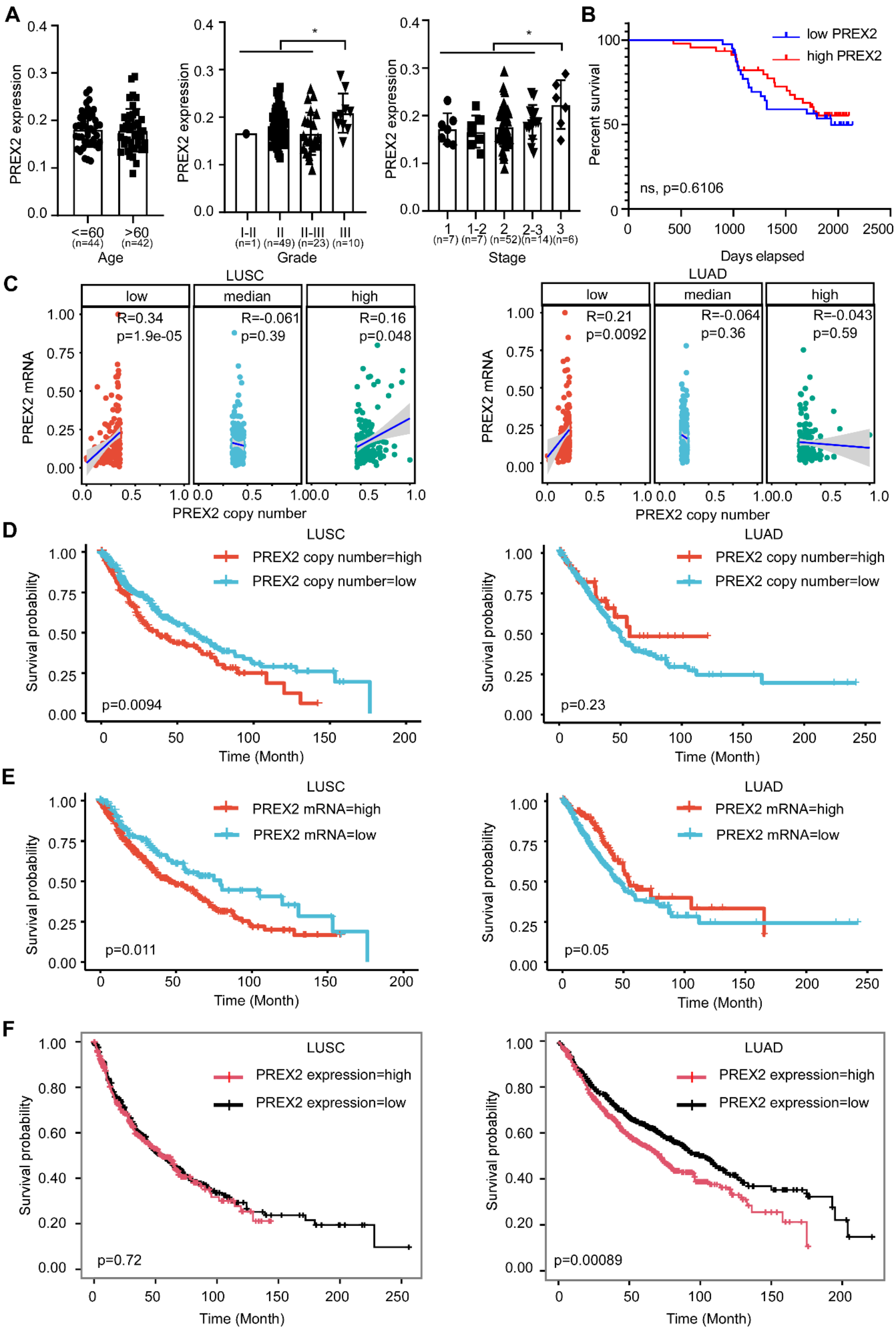
A
PREX2 expression
Age
<=60 (n=44)
>60 (n=42)
Grade
I-II (n=1)
II (n=49)
II-III (n=23)
III (n=10)
Stage
1 (n=7)
1-2 (n=7)
2 (n=52)
2-3 (n=14)
3 (n=6)
B
Percent survival
low PREX2
high PREX2
ns, p=0.6106
Days elapsed
C
LUSC
low
median
high
R=0.34 p=1.9e-05
R=-0.061 p=0.39
R=0.16 p=0.048
PREX2 mRNA
PREX2 copy number
LUAD
R=0.21 p=0.0092
R=-0.064 p=0.36
R=-0.043 p=0.59
D
LUSC
PREX2 copy number=high
PREX2 copy number=low
Survival probability
p=0.0094
Time (Month)
LUAD
p=0.23
E
LUSC
PREX2 mRNA=high
PREX2 mRNA=low
p=0.011
LUAD
p=0.05
F
LUSC
PREX2 expression=high
PREX2 expression=low
p=0.72
LUAD
p=0.00089

Figure S1. The analysis of PREX2 expression in lung cancer tissue array and lung cancer database.

A. The expression of PREX2 in patients with different ages, clinical stages and tumor grades were compared using data obtained from commercial lung cancer tissue array. Data represent means ±SD. Statistical significance was determined using student's t-test. *, $p < 0.05$.
B. The relationship between PREX2 expression level and survival probability was analyzed by Kaplan–Meier analysis using lung cancer tissue array data. ns, no significance.
C. The correlation of PREX2 copy number and mRNA was analyzed with the TCGA database.
D. The relationship between PREX2 copy number and survival probability was analyzed by Kaplan–Meier analysis using data obtained from the TCGA database.
E. The relationship between PREX2 expression and survival probability was analyzed by Kaplan–Meier analysis using transcriptome data obtained from the TCGA database.
F. The relationship between PREX2 expression and survival probability was analyzed by Kaplan–Meier analysis using the Kaplan-Meier Plotter (https://kmplot.com/).

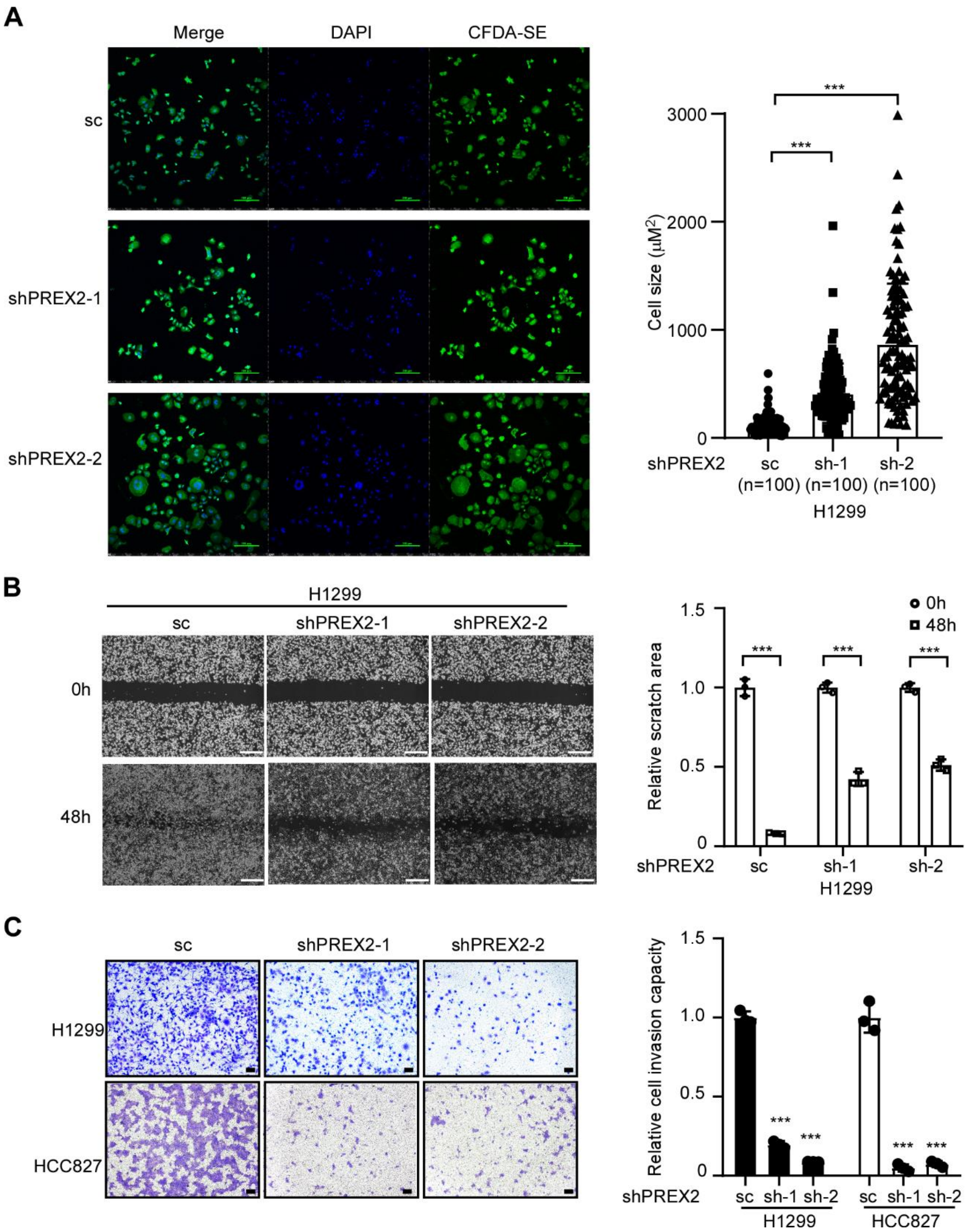

Figure S2. PREX2 knockdown affected the cell size, cell migration and invasion of NSCLC cells.

A. H1299 cells with PREX2 knockdown and control cells were stained with CFDA-SE and DAPI. The representative images are shown in the left panels. Scale bar:200 μm. The cell size was measured with ImageJ software (n=100 in each group).

B. The cell migration capacity of H1299 cells with PREX2 knockdown and control cells were evaluated by scratch wound healing assay. Time-dependent representative images are shown. Scale bar:1mm. The area of the scratch was measured with ImageJ software (n=3).
C. The cell invasion capacity of H1299 cells with PREX2 knockdown and control cells were evaluated by transwell assay. After seeding 48h, cells across the membrane were stained with 0.5% crystal violet solution, imaged using a microscope and measured with ImageJ software. Scale bar:100 μm. (n=3)
Data represent means ±SD. Statistical significance was determined using two-tailed student's t test. ***, $p < 0.001$.

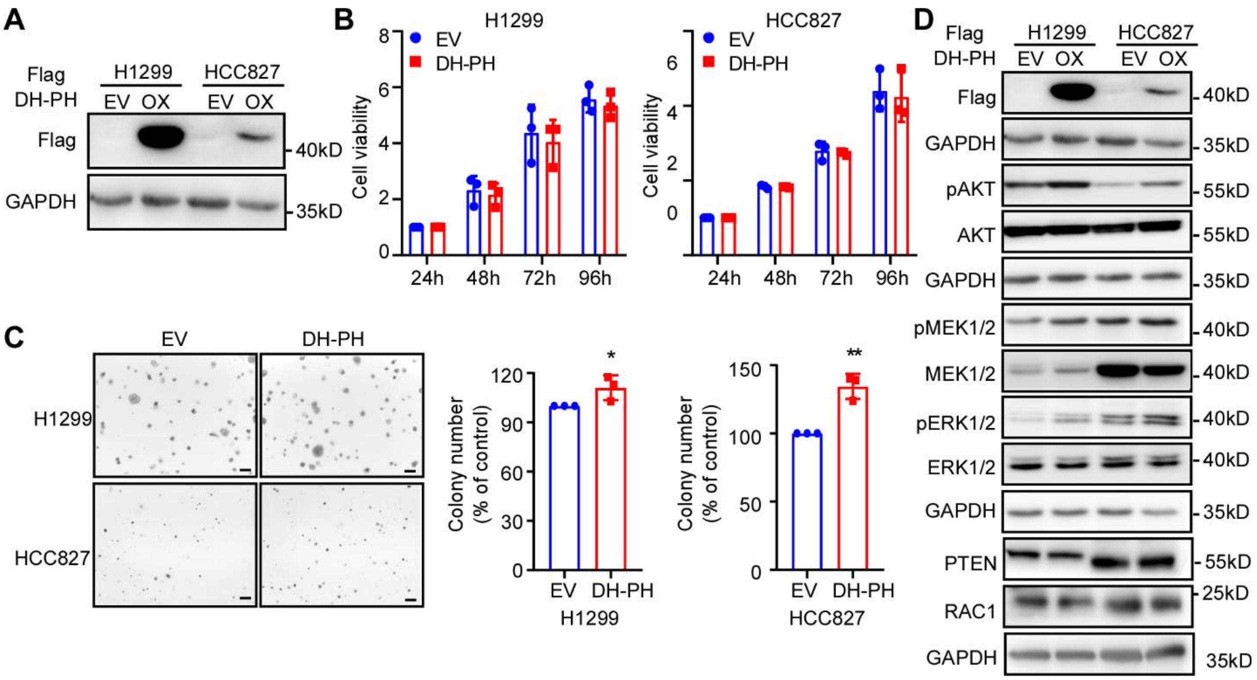

Figure S3. Overexpression of PREX2 DH-PH domain promoted the anchorage-independent growth of NSCLC cells.

A. PREX2 DH-PH domain overexpression in NSCLC cell lines was determined by western blotting. EV, empty vector. OX, overexpression.

B. Cell viability of NSCLC cells with PREX2 DH-PH domain overexpression was determined after cell seeding for 24, 48, 72 and 96 h by MTT assay. Data are presented as means ± SD from 3 independent experiments (n=3).

C. Anchorage-independent growth of NSCLC cells with PREX2 DH-PH domain overexpression was analyzed by soft agar assay. Representative images are shown in the left panels. Colonies with dimeter more than 50 μm were counted using Image-Pro Plus (v.6) computer software and normalized with control group. Data are presented as means ± SD from 3 independent experiments (n=3). The statistical analysis was determined using one-way ANOVA. Scale bar: 200 μm. *, $p < 0.05$.

D. pAKT (Ser473), pMEK1/2 (Ser217/221), and pERK1/2 (Thr202/Tyr204) were detected by western blotting in control and PREX2 DH-PH overexpression H1299 and HCC827 cells.

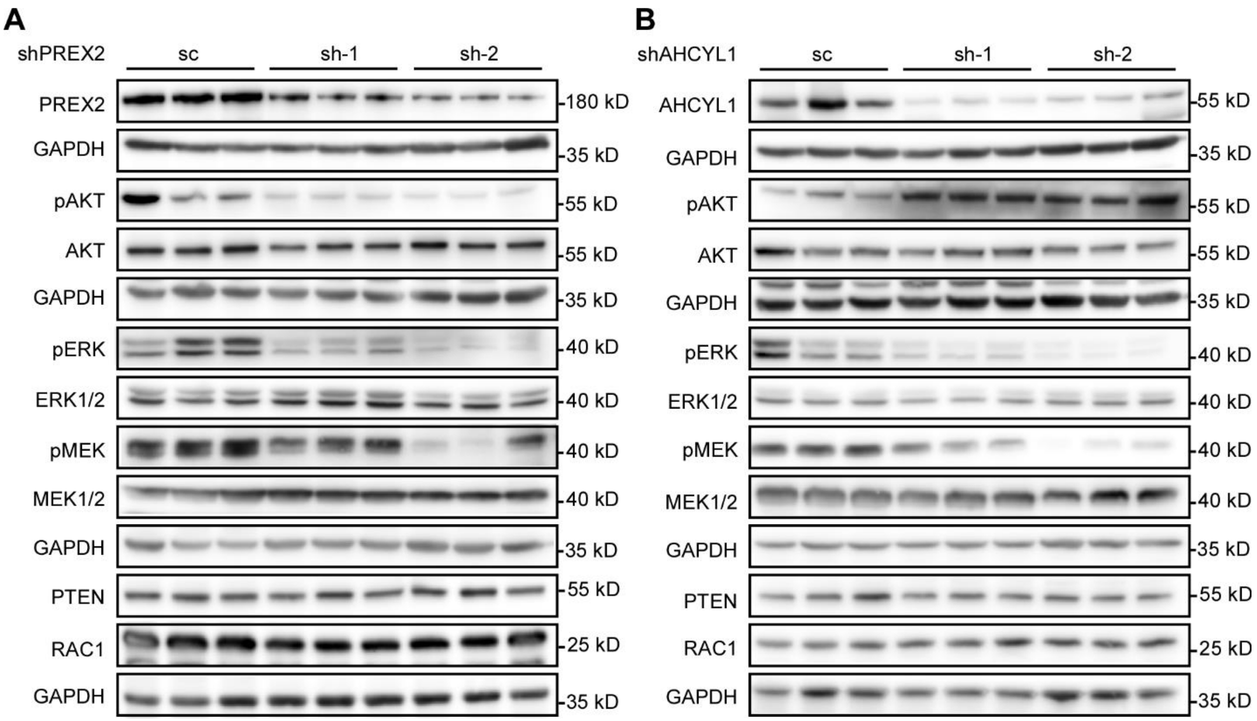

Figure S4. The MEK/ERK and PI3K/AKT signaling pathways were affected in CDX mice model.

A. The phosphorylation of AKT, MEK1/2 and ERK1/2 in tumors collected from PREX2 knockdown HCC827 cells and control cells injected mice were detected by western blotting.
B. The phosphorylation of AKT, MEK1/2 and ERK1/2 in tumors collected from AHCYL1 knockdown HCC827 cells and control cells injected mice were detected by western blotting.

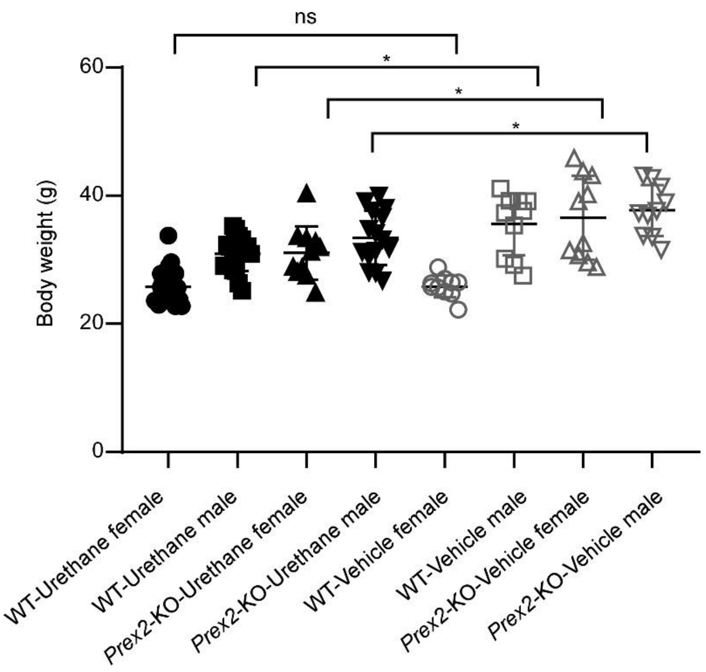

Figure S5. The body weight of vehicle and urethane-treated mice.

The body weight of urethane-treated mice was compared with vehicle-treated mice. The statistical analysis was determined using one-way ANOVA. *, $p<0.05$.

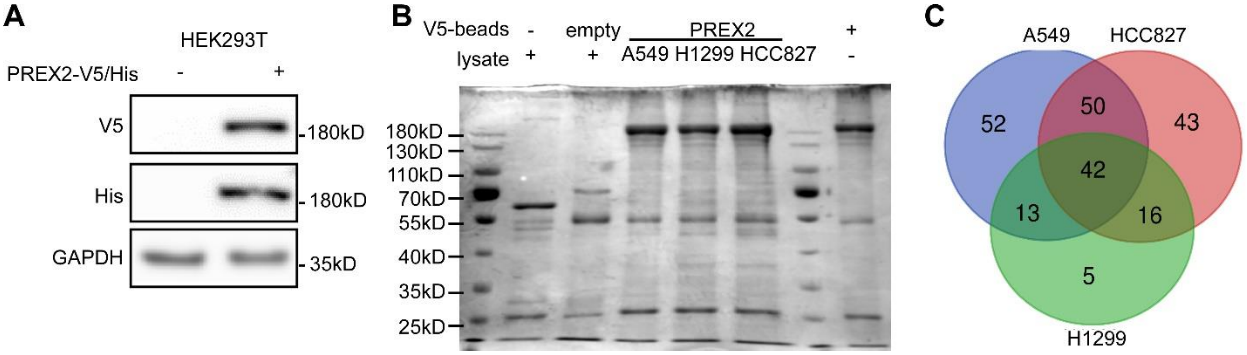

Figure S6. The identification of novel PREX2- interacting proteins by IP-MS.

A. PREX2-V5/His was transfected into HEK293T cells and western blotting was performed by anti-V5 and anti-His antibodies to make sure the expression of PREX2.
B. The cell lysates of NSCLC cell lines A549, H1299 and HCC827 were incubated with V5 beads conjugated with PREX2 overnight, and the immunoprecipitated samples were separated by SDS-PAGE gel, stained with commissive blue.
C. Venn diagram (Venny 2.1) of potential PREX2-interacting protein candidates.

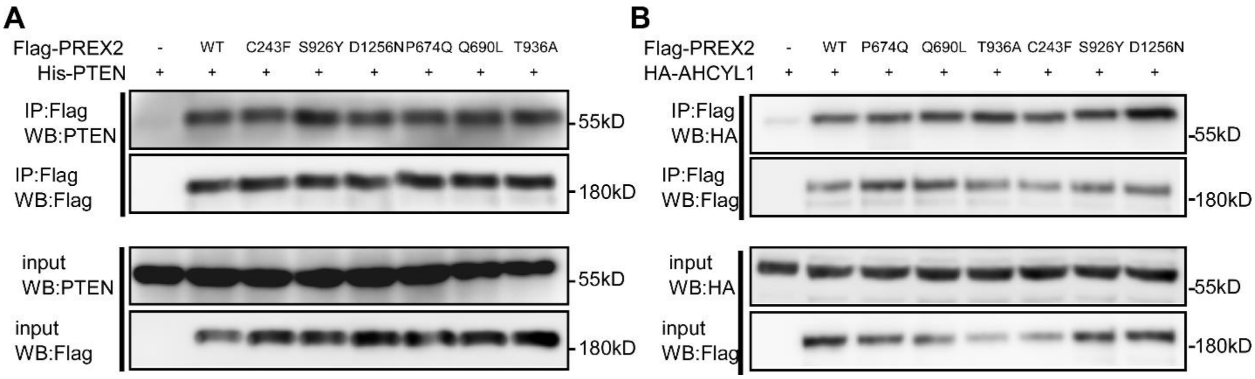

Figure S7. The interaction of PREX2 mutants with PTEN and AHCYL1 in HEK293T cells. The co-immunoprecipitations PTEN (**A**) and AHCYL1 (**B**) with PREX2 mutants were performed in HEK293T cells with flag antibody and detected by western blotting with PTEN antibody and HA antibody respectively.

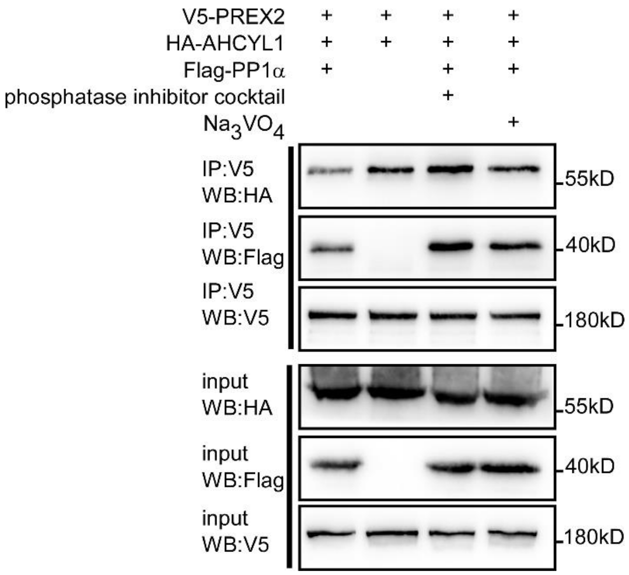

Figure S8. The active PP1α inhibits the interaction between PREX2 and AHCYL1.
Co-immunoprecipitation of HA-AHCYL1 with V5-PREX2 was performed with the presence of Flag-PP1α treated with DMSO, phosphatase inhibitor cocktail and Na_3VO_4.

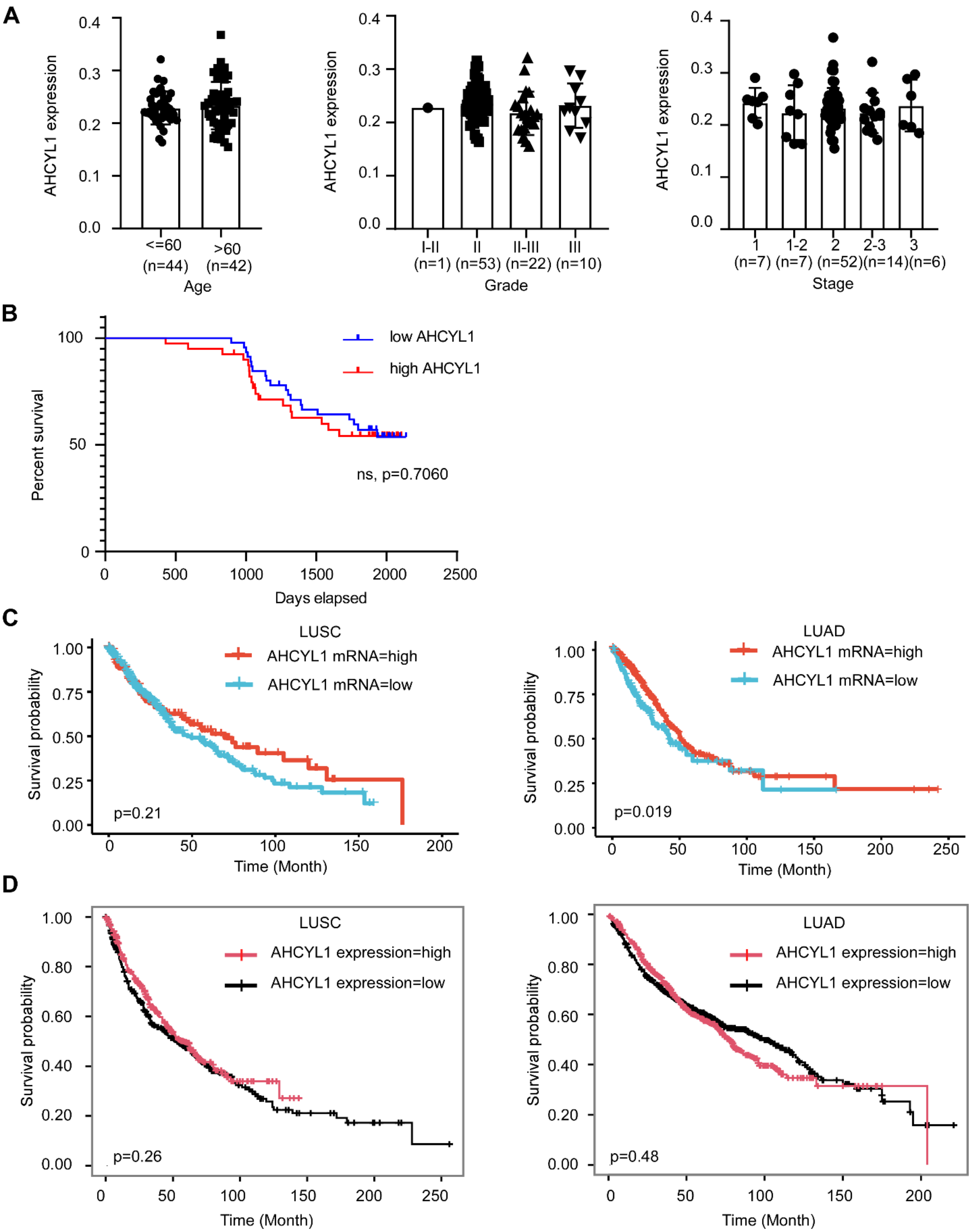
A
AHCYL1 expression
<=60 (n=44)
>60 (n=42)
Age
I-II (n=1)
II (n=53)
II-III (n=22)
III (n=10)
Grade
1 (n=7)
1-2 (n=7)
2 (n=52)
2-3 (n=14)
3 (n=6)
Stage
B
low AHCYL1
high AHCYL1
Percent survival
ns, p=0.7060
Days elapsed
C
LUSC
AHCYL1 mRNA=high
AHCYL1 mRNA=low
Survival probability
p=0.21
Time (Month)
LUAD
AHCYL1 mRNA=high
AHCYL1 mRNA=low
p=0.019
Time (Month)
D
LUSC
AHCYL1 expression=high
AHCYL1 expression=low
Survival probability
p=0.26
Time (Month)
LUAD
AHCYL1 expression=high
AHCYL1 expression=low
p=0.48
Time (Month)

Figure S9. The analysis of AHCYL1 expression in lung cancer tissue array and TCGA database.

A. The expression of AHCYL1 in patients with different ages, clinical stages and tumor grades were compared using data obtained from commercial lung cancer tissue array.
B. The relationship between AHCYL1 expression level and survival probability was analyzed by Kaplan–Meier analysis using lung cancer tissue array data. ns, no significance.
C. The relationship between AHCYL1 expression and survival probability was analyzed by Kaplan–Meier analysis using data obtained from the TCGA database.
D. The relationship between AHCYL1 expression and survival probability was analyzed by Kaplan–Meier analysis using the Kaplan-Meier Plotter (https://kmplot.com/).

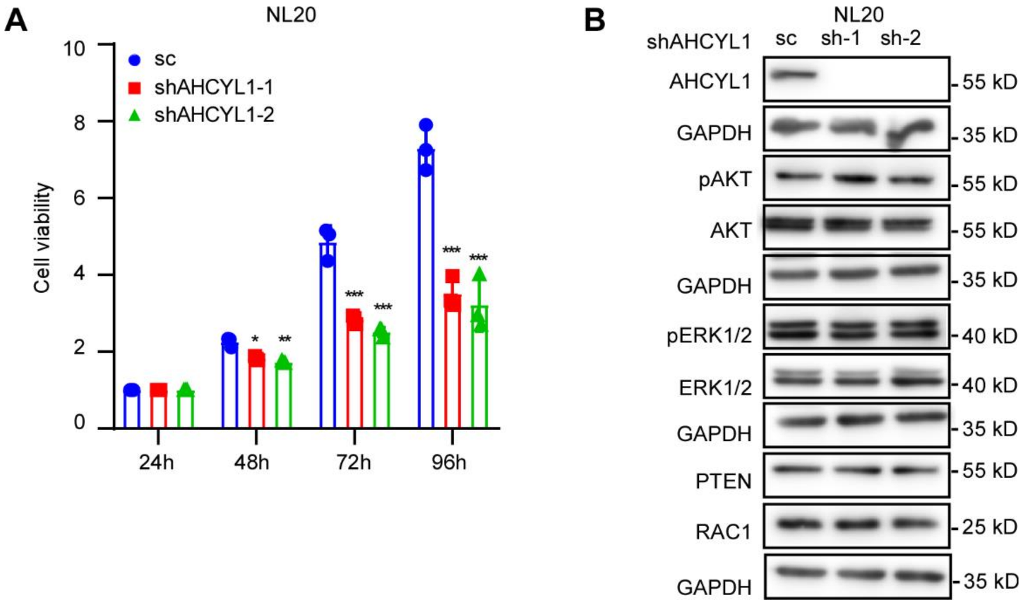

Figure S10. Knockdown of AHCYL1 suppressed the cell growth of NL20.

A. Cell viability of AHCYL1 knockdown NL20 after cell seeding for 24, 48, 72 and 96 h was determined by MTT assay. Data are presented as means ± SD from 3 independent experiments (n = 3). The statistical analysis was determined using multiple t test. *, $p < 0.05$. **, $p < 0.01$. ***, $p < 0.001$.

B. pAKT (Ser473), pMEK1/2 (Ser217/221), and pERK1/2 (Thr202/Tyr204) were detected by western blotting in AHCYL1 knockdown NL20 and control cells.

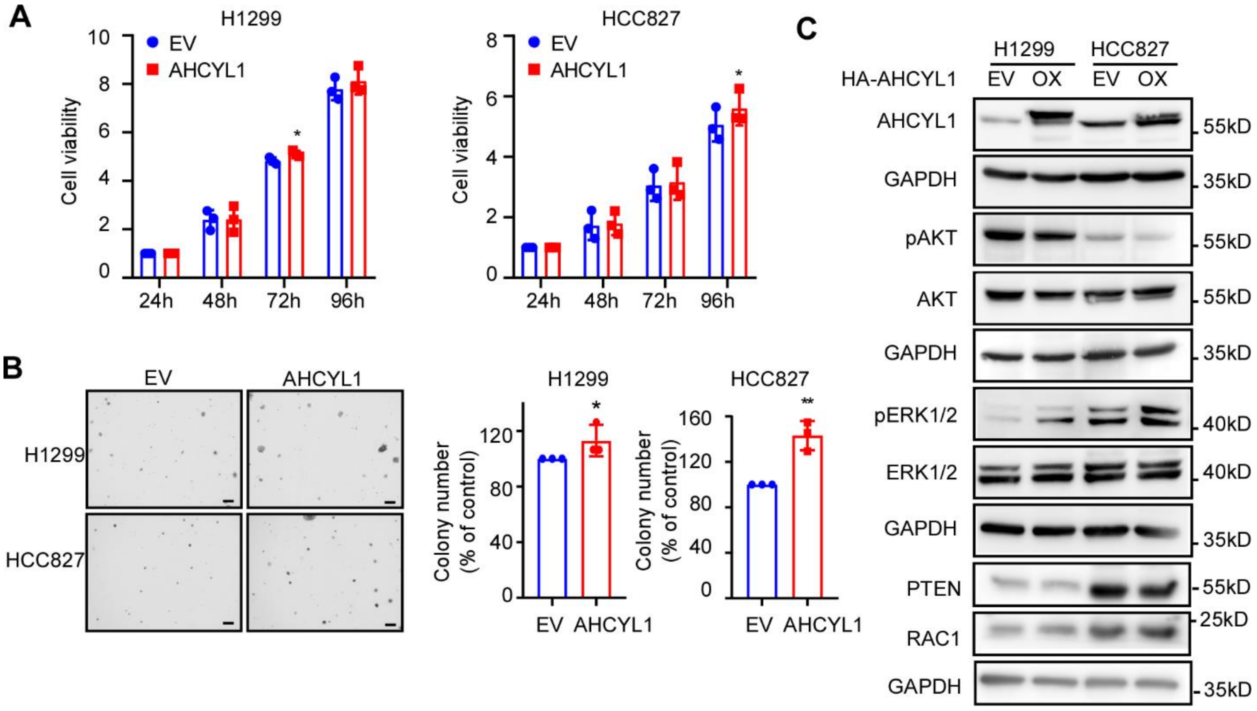

Figure S11.Overexpression of AHCYL1 promoted the anchorage-independent growth of NSCLC cells.

A. Cell viability of NSCLC cells with AHCYL1 overexpression was determined after cell seeding for 24, 48, 72 and 96 h by MTT assay. Data are presented as means ± SD from 3 independent experiments (n = 3). The statistical analysis was determined using multiple t test. *, $p < 0.05$.

B. Anchorage-independent growth of NSCLC cells with AHCYL1 overexpression was analyzed by soft agar assay. Representative images are shown in the left panels. Colonies with dimeter more than 50 μm were counted using Image-Pro Plus (v.6) computer software and normalized with control group. Data are presented as means ± SD from 3 independent experiments. The statistical analysis was determined using one-way ANOVA. Scale bar: 200 μm. *, $p < 0.05$. **, $p < 0.01$.

C. pAKT (Ser473), pMEK1/2 (Ser217/221), and pERK1/2 (Thr202/Tyr204) were detected by western blotting in control and AHCYL1 overexpression H1299 and HCC827 cells.

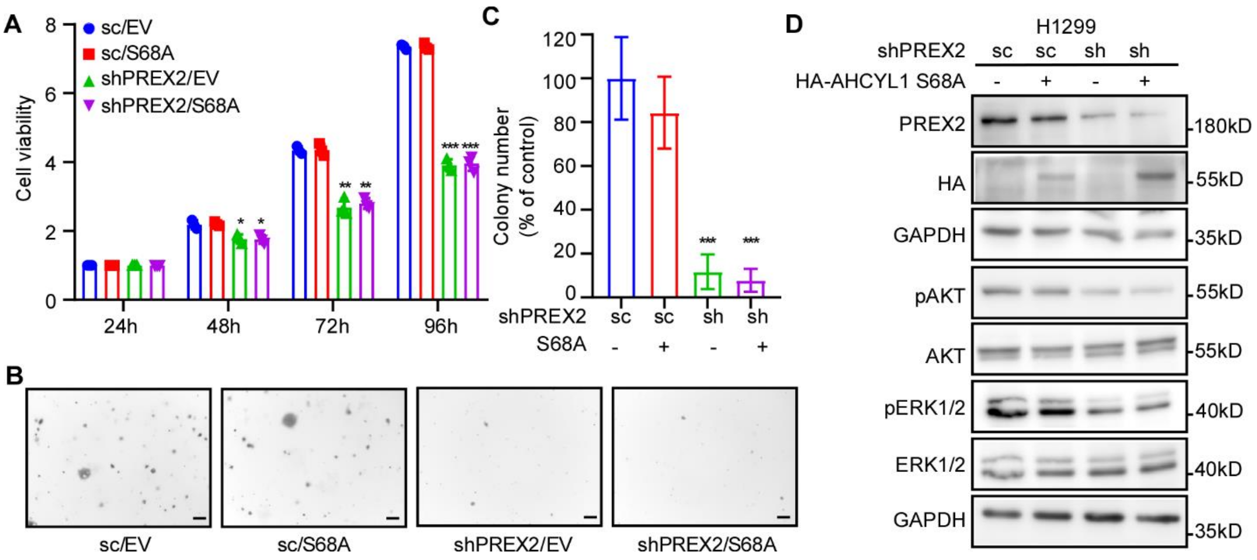

Figure S12.

Overexpression of AHCYL1 S68A could not rescue the cell growth inhibition caused by PREX2 knockdown in H1299.

A. The cell viability of H1299 with AHCYL1 S68A overexpression upon PREX2 knockdown was assessed by MTT assay. Data are presented as means ± SD from 3 independent experiments (n = 3). The statistical analysis was determined using multiple t test. *, $p < 0.05$. **, $p < 0.01$. ***, $p < 0.001$.

B and **C** Anchorage-independent growth of H1299 with AHCYL1 S68A overexpression upon PREX2 knockdown was detected by soft agar assay. The representative images are shown in (B). Scale bar: 200 μm. The statistical analysis was determined using one-way ANOVA. ***, $p < 0.001$.

D. AKT and ERK1/2 phosphorylation were detected by western blotting in H1299 with AHCYL1 S68A overexpression upon PREX2 knockdown.

Table S1. Primers used in paper

Construction		Primer Sequence (5'→3')
Flag-PREX2	F	CGGGATCCAACCATGGATTACAAGGATGACGACGATAAGATGAGCGAGGACAGCCGC
	R	CCGCTCGAGCCTTCTTCTCCAGCTGGGGGAGG
Flag-PREX2DHPH	F	CGGGATCCAACCATGGATTACAAGGATGACGACGATAAGATGAGCGAGGACAGCCGC
	R	CCGCTCGAGTCAGCACATCATTTTATAAAGTTT
DHPH-OX	F	GCTCTAGAACCATGGATTACAAGGATGACGACGATAAG
	R	AGAAGCGGCCGCTTAACCTTTCCGCCGTTCTCTTT
AHCYL1-OX	F	GCTCTAGA ATGTATCCTTACGACGTGCCT
	R	CGGGATCC TCAGTATCTGTAATAATTAGG
shPREX2-1	F	CCGGCGAATTTGTGTCATGGCTGTTCTCGAGAACAGCCATGACACAAATTCG TTTTTG
	R	AATTCAAAAACGAATTTGTGTCATGGCTGTTCTCGAGAACAGCCATGACACAAATTCG
shPREX2-2	F	CCGGGAACAGGGTGAGAAACTTTATCTCGAGATAAAGTTTCTCACCCTGTTC TTTTTG
	R	AATTCAAAAAGAACAGGGTGAGAAACTTTATCTCGAGATAAAGTTTCTCACCCTGTTC
shAHCYL1-1	F	CCGGCGGCAAGTCGATGTCGTAATACTCGAGTATTACGACATCGACTTGCCGTTTTTG
	R	AATTCAAAAACGGCAAGTCGATGTCGTAATACTCGAGTATTACGACATCGACTTGCCG
shAHCYL1-2	F	CCGGCAATGTCTAAATCGCCTTAAACTCGAGTTTAAGGCGATTTAGACATTGTTTTTG
	R	AATTCAAAAACAATGTCTAAATCGCCTTAAACTCGAGTTTAAGGCGATTTAGACATTG
S68A	F	CAGTGGAGGACTGTGCGATCGAGCGAGACAA
	R	TTGTCTCGCTCGATCGCACAGTCCTCCACTG
I42A/F44A (2A)	F	CTGCATGTCATCAGCAGCCTGGGCTTGCTTCTTGGGCGCCTTGGTG
	R	CACCAAGGCGCCCAAGAAGCAAGCCCAGGCTGCTGATGACATGCAG
R96A	F	AAGGACTGCTTGCCCGCGTCCACATAGTCCCC
	R	GGGGACTATGTGGACGCGGGCAAGCAGTCCTT

GST-AHCYL1	F	CG GGATCC ATGTCGATGCCTGACGCGATG
	R	GCCTCGAG TCAGTATCTGTAATAATTAGG
GST-AHCYL1	F	CG GGATCC ATGTCGATGCCTGACGCGATG
	R	GCCTCGAG TCAGTATCTGTAATAATTAGG
Myc-DH (aa23-214)	F	CTCGAGTACTGCGCGTGTGCGTGCTCAG
	R	GCGGCCGCTTAGGCCTCGTTTATGTTGGAAC
Myc-PH (aa245-365)	F	CTCGAGGGGAAATGCTAATGTGTGGAGTCT
	R	GCGGCCGCTTAACCTTTCCGCCGTTCTCTTT
Myc-DEP1 (aa390-465)	F	CTCGAGGATGCAGACAAGGAAATCTGATCA
	R	GCGGCCGCTTAATCATAGCGAAATCTATATAAC
Myc-DEP2 (aa489-566)	F	CTCGAGGGCATAGCCTTTTTACTCCAGTGATAAG
	R	GCGGCCGCTTAATCCGAAAAAAAACGGAAAAGT
Myc-PDZ1 (aa591-673)	F	CCGCTCGAGGGAAGTCATTATTGATTAAATCCAAT
	R	GCGGCCGCTTACTTTGTGCTCACAAGAACT
Myc-PDZ2 (aa675-758)	F	CTCGAGGGAGAGAGACAGTGAAAATTCCAGAT
	R	AGAAGCGGCCGCTTAATAAACCCATTGTATGGAAT
Myc-PDZ (aa592-758)	F	CCGCTCGAGGGAAGTCATTATTGATTAAATCCAAT
	R	AGAAGCGGCCGCTTAATAAACCCATTGTATGGAAT
HA-AHCYL1(1-104aa)	F	GGGGTACC ATGTATCCTTACGACGTGCCT
	R	GCTCTAGA TTA ATTGCTGCTGCCCTTGGAG
HA-AHCYL1(104-530aa)	F	GGGGTACCATGTATCCTTACGACGTGCCTGACTACGCCAATTTCT GTGTGAAGAACA
	R	GCTCTAGA TCAGTATCTGTAATAATTAGG

HA-AHCYL1(1-138aa)	F	GGGGTACC ATGTATCCTTACGACGTGCCT
	R	GCTCTAGA TTA CTGAGCACGTTTCCTGAGTGA
HA-AHCYL1(1-169aa)	F	GGGGTACC ATGTATCCTTACGACGTGCCT
	R	GCTCTAGA TTA CCCCAGGGCACAGAGTGTCTC
HA-AHCYL1(1-201aa)	F	GGGGTACC ATGTATCCTTACGACGTGCCT
	R	GCTCTAGA TTA CTTCCAAGCGAACACTGCAAC
HA-AHCYL1(1-231aa)	F	GGGGTACC ATGTATCCTTACGACGTGCCT
	R	GCTCTAGA TTA TCCCCCATCATCCAGGATCAT
HA-AHCYL1(1-526aa)	F	GGGGTACC ATGTATCCTTACGACGTGCCT
	R	GCTCTAGA TTA ATTAGGTTTGAATGGCCCA
HA-AHCYL1(1-520aa)	F	GGGGTACC ATGTATCCTTACGACGTGCCT
	R	GCTCTAGA TTA ATTTTTGTTGAGTCCCAGATA
HA-AHCYL1(138-530aa)	F	GGGGTACCATGTATCCTTACGACGTGCCTGACTACGCCCAGGGG GAGAAGCCCTTGGC
	R	GCTCTAGA TCAGTATCTGTAATAATTAGG
HA-AHCYL1(201-530aa)	F	GGGGTACCATGTATCCTTACGACGTGCCTGACTACGCCAAGGGC GAGTCAGAAGATGA
	R	GCTCTAGA TCAGTATCTGTAATAATTAGG
HA-AHCYL1(231-530aa)	F	GGGGTACCATGTATCCTTACGACGTGCCTGACTACGCCGGAGAC TTAACCCACTGGGT
	R	GCTCTAGA TCAGTATCTGTAATAATTAGG
WT/PREX2 KO	F	CTCCAGTTATGTCTGTTTGCTTGAG
WT	R	CCAATCCCTAGCTCCCTCTTTCC
PREX2 KO	R	GCACACACATTCCAGGTCAAC

Tables S2. 42 overlapped PREX2-interacting proteins

Reference		Name
P15880	RS2	40S ribosomal protein S2
P61247	RS3A	40S ribosomal protein S3a
P62701	RS4X	40S ribosomal protein S4, X isoform
P62753	RS6	40S ribosomal protein S6
P10809	CH60	60 kDa heat shock protein, mitochondrial
P26373	RL13	60S ribosomal protein L13
P36578	RL4	60S ribosomal protein L4
Q02878	RL6	60S ribosomal protein L6
P62424	RL7A	60S ribosomal protein L7a
P63261	ACTG	Actin, cytoplasmic 2
**O43865**	**SAHH2**	**Adenosylhomocysteinase like 1**
Q08211	DHX9	ATP-dependent RNA helicase A
P13639	EF2	Elongation factor 2
P49411	EFTU	Elongation factor Tu, mitochondrial
Q99613	EIF3C	Eukaryotic translation initiation factor 3 subunit C
O15371	EIF3D	Eukaryotic translation initiation factor 3 subunit D
P04406	G3P	Glyceraldehyde-3-phosphate dehydrogenase
P07900	HS90A	Heat shock protein HSP 90-alpha
P52597	HNRPF	Heterogeneous nuclear ribonucleoprotein F
Q00839	HNRPU	Heterogeneous nuclear ribonucleoprotein U
P22626	ROA2	Heterogeneous nuclear ribonucleoproteins A2/B1
P16402	H13	Histone H1.3
Q4FZB7	SV421	Histone-lysine N-methyltransferase SUV420H1
Q86YZ3	HORN	Hornerin
P06314	KV404	Ig kappa chain V-IV region B17
P13645	K1C10	Keratin, type I cytoskeletal 10
P02533	K1C14	Keratin, type I cytoskeletal 14
P08779	K1C16	Keratin, type I cytoskeletal 16
P35527	K1C9	Keratin, type I cytoskeletal 9
P04264	K2C1	Keratin, type II cytoskeletal 1
P35908	K22E	Keratin, type II cytoskeletal 2 epidermal
P13647	K2C5	Keratin, type II cytoskeletal 5
P02538	K2C6A	Keratin, type II cytoskeletal 6A
P00338	LDHA	L-lactate dehydrogenase A chain
P07195	LDHB	L-lactate dehydrogenase B chain
P19338	NUCL	Nucleolin
P06748	NPM	Nucleophosmin
Q70Z35	PREX2	Phosphatidylinositol 3,4,5-trisphosphate-dependent Rac
P00558	PGK1	Phosphoglycerate kinase 1
Q9NQH7	XPP3	Probable Xaa-Pro aminopeptidase 3
Q5VTE0	EF1A3	Putative elongation factor 1-alpha-like 3
P14618	KPYM	Pyruvate kinase PKM
